# Multivalent DNA Origami Enables Single-Molecule Dissection of Integrin αvβ6–Receptor Tyrosine Kinase Crosstalk in Cancer Biology

**DOI:** 10.1021/acsnano.5c07581

**Published:** 2025-08-26

**Authors:** Tingting Zheng, Lauren Grace Rigby, John F. Marshall, Matteo Palma

**Affiliations:** † Department of Chemistry, 4617 Queen Mary University of London, Mile End Road, London E1 4NS, United Kingdom; ‡ Barts Cancer Institute, Cancer Research UK Centre of Excellence, Queen Mary University of London, Charterhouse Square, London EC1M 6BQ, United Kingdom

**Keywords:** DNA origami, nanoscale ligand patterning, single-molecule control, multivalent interactions, receptor tyrosine kinase, integrin αvβ6, cancer cell signaling

## Abstract

Nanoscale organization of integrin-mediated receptor crosstalk is crucial for controlling cellular signaling in cancer biology. Previously, interactions between integrin αvβ6 and receptor tyrosine kinases (RTKs) have been implicated in cancer progression, but the spatial regulatory mechanisms remain undefined. Here, we developed a programmable DNA origami-based platform for nanoscale control of heteroligand multivalency and spacing, enabling systematic investigation of αvβ6–RTK interactions in cancer biology. We identified a spatial activation threshold for the αvβ6-specific peptide A20FMDV2 that promotes A375P β6 cell adhesion and FAK phosphorylation along with spacing- and density-dependent EGFR phosphorylation triggered by EGFR aptamers. Importantly, at an optimized peptide-to-RTK (EGFR, HER2, and Met) aptamer ratio and ligand density, αvβ6–RTK coactivation synergistically enhanced cell spreading and amplified phosphorylation of AKT and ERK, part of the PI3K–AKT and Ras–MAPK pathways. Validation in breast cancer models (MDA-MB-468 and BT-474) highlighted cell-type-specific signaling dependencies. This platform offers a framework for tumor microenvironment mimics and integrin–RTK-targeted therapies, emphasizing the critical role of nanoscale ligand patterning and multivalency in cancer progression.

## Graphical abstract

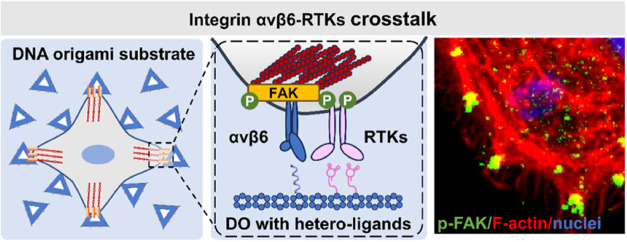

Integrins are transmembrane proteins crucial in the regulation of cellular behavior via their interaction with the extracellular matrix (ECM).^1–4^ Upregulation of the epithelial-specific integrin αvβ6 has been associated with worse overall survival rates in several malignancies.^5–7^ This is due to the role of αvβ6 in promoting cancer cell migration, invasion, and carcinogenesis, processes that are partially reliant on receptor tyrosine kinase (RTK) activation. Specifically, αvβ6 integrin interacts with RTK signaling pathways, driving cellular behaviors that drive tumor progression and metastasis.^8,9^ Despite significant advances in understanding integrin–RTK interactions,^10^ the precise spatial organization, stoichiometric relationships, and cooperative signaling mechanisms underlying their shared pathways in carcinogenesis remain poorly characterized. Furthermore, the spatial activation thresholds of these ligand–receptor systems and their functional implications require systematic investigation. However, studying nanoscale interactions and their multivalent cooperation remains a major challenge in cancer biology, as existing methods lack precise control over integrin–RTK crosstalk within a unified platform.

Different approaches have been used to control the spatial organization of cellular adhesion receptors for the fabrication of biomimicking surfaces,^11–19^ with the use of ligand-anchoring metal nanodots being the most significant in terms of nanoscale spatial resolution. In this regard, micellar diblock copolymer self-assembly has been successful in demonstrating the importance of nanoscale clustering of integrins,^13–15^ but it does not allow for precise stoichiometric and multivalent control. Differently, lithography-based techniques have been noteworthy for the investigation of multivalent interactions in cell membranes; however, the time-consuming and relative complex top-down fabrication process (*e.g.*, to present distinct metal dots for different ligands) typically constrains their ease of use and design, hence partly limiting their broad applicability.^20–22^

Notably, bottom-up nanofabrication strategies based on the use of DNA nanostructures (origami)^23^ enable nanoscale-precise positioning of bioactive ligands with tunable spatial resolution (∼6 nm)^23–27^ and multivalent single-molecule control.^27–38^ Hence, DNA origami has emerged as a powerful tool to investigate the link between multivalent ligand spatial information and cellular behavior, remarkably also for the regulation of receptor-mediated signaling, including EphA2,^27,39^ Fc receptors (FcRs),^40^ TLR9,^41^ Met,^42^ CD95,^43^ insulin receptors (IRs),^44^ Jag1,^45^ T cell receptors (TCRs),^46–50^ and B-cells.^30^ However, the potential of DNA nanostructures to engineer heteromultivalent ligand systems for probing receptor crosstalk remains underexplored. This platform enables the systematic examination of different synergistic interactions, including integrin αvβ6–RTK cooperativity and its regulation of cell adhesion, which are of particular interest.

Herein, we employed a DNA origami-based platform to present heteromultivalent ligands for the investigation of integrin αvβ6 and RTK copresentation, with single-molecule resolution and nanoscale spatial control (Figure 1a). This allowed us to demonstrate (i) a spatial activation threshold for melanoma cell (A375P β6) spreading, defined by the required density and nanoscale spacing of the αvβ6-specific peptide A20FMDV2; (ii) aptamer-triggered RTK phosphorylation modulation by spatial proximity; and (iii) synergistic signaling amplification, whereby integrated integrin αvβ6 and RTKs promote cell spreading and coactivate FAK and RTKs, leading to the activation of PI3K–AKT and Ras–MAPK/ERK pathways. We further confirmed our results in breast cancer models, revealing cell-type-specific integrin–RTK crosstalk influenced by RTK and integrin expression levels. The platform we developed allows for high precision in mimicking the tumor microenvironment, in particular, for the systematic dissection of multivalent interactions governing cancer cell spreading and signaling pathway activation.

**Figure 1:**
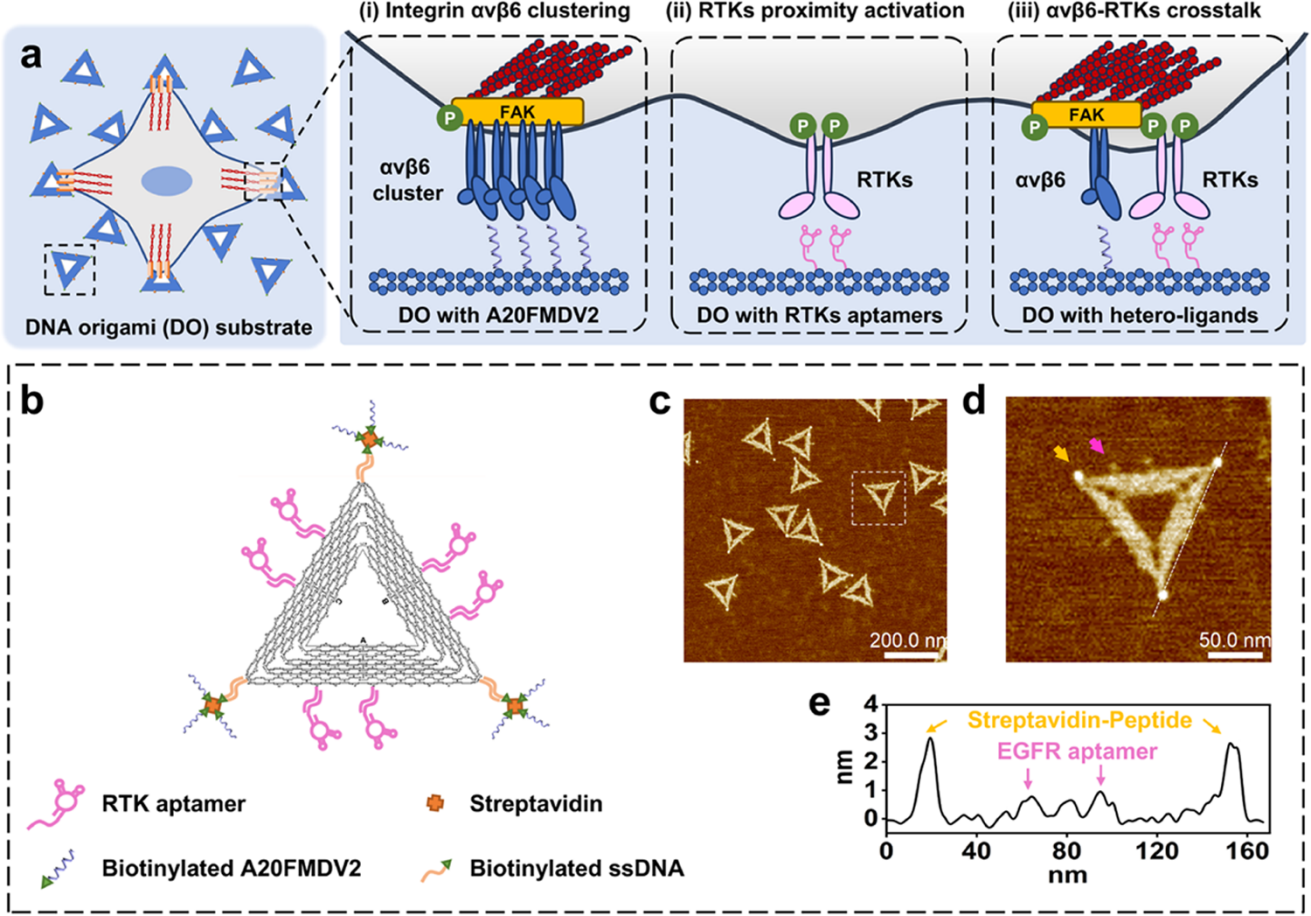
Fabrication of DNA origami for spatially patterned ligands to regulate receptor organization and signal activation. (a) Schematic illustration of DNA origami directing ligand spatial distribution to regulate membrane receptor organization and cell spreading behavior: (i) αvβ6 clustering activates FAK phosphorylation, (ii) RTK proximity induces phosphorylation, and (iii) αvβ6–RTK crosstalk. (b) Strategy for functionalizing DNA origami with EGFR aptamers (via sticky end hybridization) and A20FMDV2 peptides (via biotin–streptavidin binding). (c) AFM image of DNA origami functionalized with three peptides and six aptamers (DO_3PP_6E). (d) Zoom-in of a modified origami from (c); yellow arrow, streptavidin-peptide; pink arrow, EGFR aptamer. (e) Cross-section profile from the inset of (d).

## Results and Discussion

### Assembly and Characterization of Ligand-Functionalized DNA Origami

Triangular DNA origami structures^23^ were designed and synthesized with three 120 nm sides and a central triangular hole (Figure S1). DNA origami solutions cast on mica and imaged via atomic force microscopy (AFM) exhibited a height of approximately 1.5–2 nm (Figure S1a–c), consistent with the height of double-stranded DNA (dsDNA).^23^ We selected the integrin αvβ6-specific A20FMDV2 peptide^51^ and EGFR/HER2/Met aptamers^52–54^ as binding ligands, in line with our previous work demonstrating EGFR-specific ligand cooperative effect with A20FMDV2 in controlling cutaneous melanoma cell spreading behavior.^28^ A biotin–streptavidin binding strategy was employed to modify the DNA origami with A20FMDV2 peptides, while aptamers were assembled via hybridization with complementary sticky ends (Figure 1b). AFM imaging showed the ligands’ location on individual origami structures: hybridized aptamers (*e.g*., EGFR aptamers) are visible as 1 nm dots, while streptavidin-conjugated peptides appeared as larger 3 nm particles (Figure 1c–e).

To enable cancer cell attachment and spreading, functionalized DNA nanostructures were immobilized on glass coverslips using a covalent binding strategy (Figure S2). Fifteen amino anchors were placed at the origami’s center (Figure S1d) to form amide bonds with carboxylic silanized coverslips (Figure S2a). Unlike physically adsorbed DNA origami, covalently immobilized structures exhibit strong adhesion to the coverslips and greater resistance to rinsing (Figure S2b,c). An antiserum degradation test confirmed that the origami remained intact in the cell medium for at least 3 h (Figure S3), highlighting the robustness of the platform in view of subsequent cell adhesion investigations.

### Spatial Threshold for Integrin αvβ6-Mediated Cancer Cell Spreading

In this study, we used an isogenic human melanoma cell pair (A375P puro and A375P β6), differing only in integrin αvβ6 expression^28^ (Figures S4–S8), as a model to examine the organization, stoichiometry, and functional impact of integrin αvβ6-associated ligands on cell behavior. We functionalized triangular DNA origami structures with three A20FMDV2 peptides at varying intervals (30–120 nm). Ligand density was controllably increased (from 3 to 12 peptides/origami, *i.e*., from 87 ± 16 to 347 ± 65 peptides/μm^2^, as calculated from the DNA origami surface density shown in Figure S9), with topographical AFM images shown in Figures 2a and S10a,b for all designs and agarose gel analysis in Figure S10c,d, confirming successful DNA origami functionalization with the aforementioned peptides. After 1.5 h of incubation on DNA origami substrates, A375P β6 cells exhibited spreading in a spacing- and density-dependent manner, revealing a critical nanoscale threshold for integrin-mediated adhesion.

**Figure 2:**
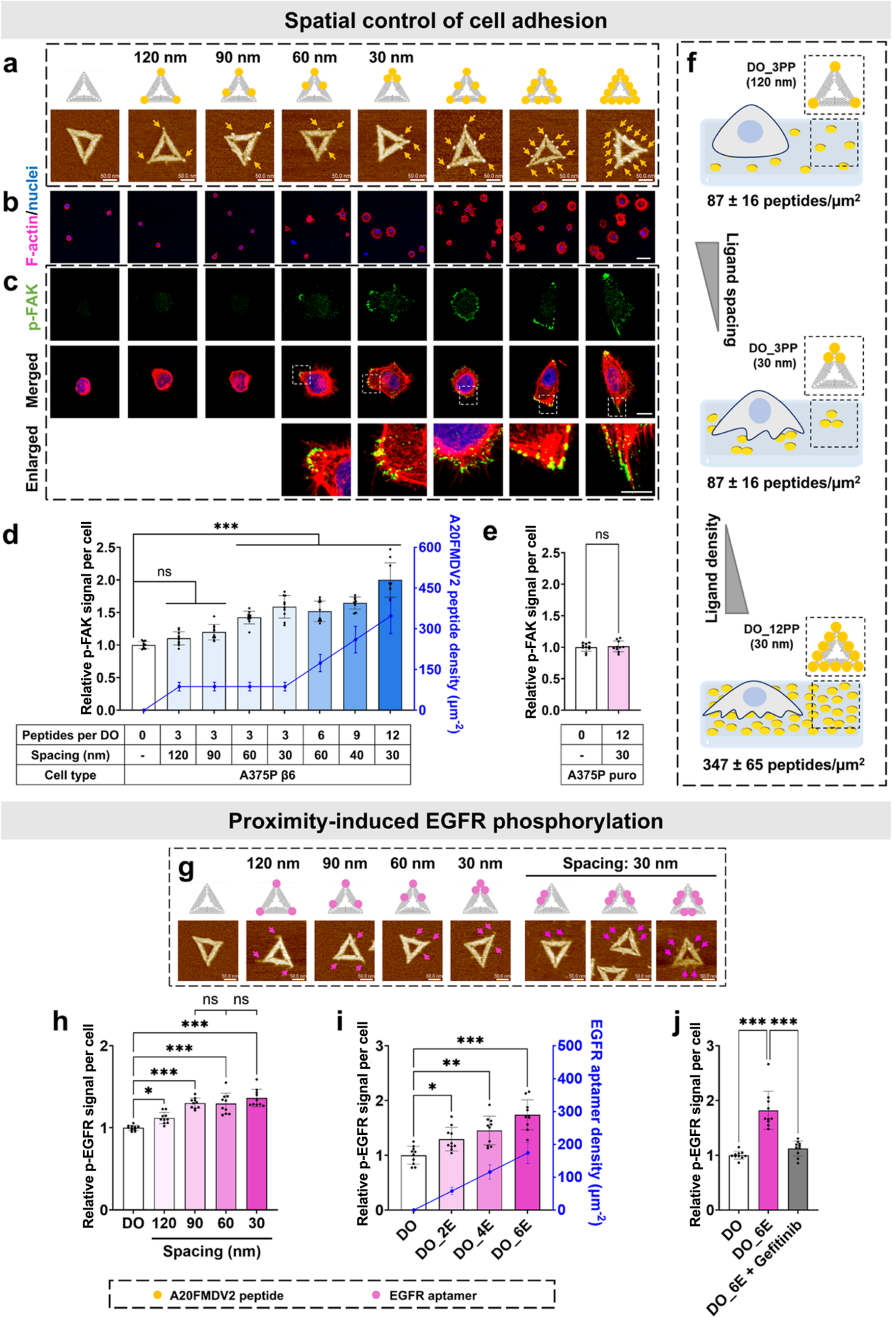
Spatially patterned ligands on DNA origami regulate A375P β6 cell spreading in a spacing- and density-dependent manner. (a) AFM images of DNA origami functionalized with A20FMDV2 peptides at defined spacings (30–120 nm, 87 ± 16 peptides/μm^2^) and densities (3–12 peptides/origami; from 87 ± 16 to 347 ± 65 peptides/μm^2^). Scale bar, 50 nm. (b) Confocal images of A375P β6 cell spreading on peptide-functionalized substrates. F-actin, red; nuclei, blue. Scale bar, 40 μm. (c) Representative Z-projected confocal images of p-FAK (Y397, green) in A375P β6 cells spreading on peptide-functionalized substrates. Scale bar: 10 μm; enlarged images’ scale bar: 20 μm. Additional representative single-cell morphologies are shown in Figure S11, and large-field overviews demonstrating population-level consistency are provided in Figure S13. (d,e) Left *Y*-axis: normalized p-FAK intensity in (d) A375P β6 and (e) A375P puro cells, with DO as the control (one-way ANOVA, *n* = 10, ***P < 0.001; ns, not significant). Right *Y*-axis: A20FMDV2 peptide density of substrates. Data represent mean ± SD. (f) Scheme showing spreading behavior regulated by peptide density and spacing. (g) Scheme and AFM images of EGFR aptamer-functionalized origami with defined spacings (30–120 nm, 87 ± 16 aptamers/μm^2^) and varied valencies (2, 4, or 6 EGFR aptamers per DNA origami; 58  ±  11, 116 ±  22, 174  ±  32 aptamers/μm^2^). Scale bar, 50 nm. (h, i) Normalized p-EGFR intensity in A375P β6 cells on substrates with various (h) aptamer spacings and (i) densities, with DO as the control. Data represent mean ± SD (one-way ANOVA, *n* = 10, *P < 0.05, **P < 0.01, ***P < 0.001; ns, not significant). (j) Gefitinib inhibits aptamers’ proximity-induced EGFR phosphorylation. Data represent mean ± SD (one-way ANOVA, *n* = 10, ***P < 0.001).

In particular, our data indicate that a density of 87 ± 16 peptides/μm^2^ (three peptides/origami) effectively promoted cell spreading when the peptide spacing was 60 nm (2.9-fold *vs* nonligand conditions, DO) or less (30 nm, 3.6-fold). In contrast, spacings of 90–120 nm failed to induce adhesion (Figures 2b, S11, and S12). The 60 and 30 nm spacing substrates promoted increased integrin clustering and focal adhesion (FA) formation, which were localized to the juxtamembrane region and colocalized with F-actin within the cell’s protrusive structures (Figure 2c; see also Figure S13 for large-field overviews showing population-level consistency). Below this threshold, *i.e.*, for spacings of 60 and 30 nm, integrin clustering and downstream focal adhesion kinase phosphorylation (p-FAK, Y397) were markedly increased, up to 1.4- and 1.6-fold respectively (Figure 2d). This spacing is consistent with the requirements for integrin αvβ6 clustering and Talin recruitment, both crucial for mechanotransduction.^15,18,55^

Moreover, the results demonstrated integrin αvβ6 binding, ligand density-dependent spreading, and p-FAK signaling. Increasing the peptide density to 347 ± 65 peptides/μm^2^ (12 peptides per origami, DO_12PP) at an optimal spacing of 30 nm further enhanced cell spreading by 4.8-fold (Figure S12) and doubled p-FAK intensity (Figure 2d) compared to DO. These effects arise from high-density, low-spacing ligand arrays, which stabilize FAs by promoting integrin clustering, enhancing the recruitment of adhesion proteins (*e.g.*, Talin and Vinculin),^11^ and optimizing force distribution to prevent adhesion collapse.^15^ Compared to the αvβ6-positive A375P β6 cells, A375P puro cells spreading on the high-density DNA origami ligand substrate (DO_12PP) showed no significant morphological changes or an increase in p-FAK levels relative to the control substrate (DO) (Figure 2e). These results highlight the importance of integrin organization in cell spreading, with integrin signaling being both ligand-density-dependent and governed by spatial cooperativity, where increased ligand density and lower spacing promote integrin clustering, FAK activation, and cytoskeletal remodeling (Figure 2f).

### Proximity-Induced EGFR Phosphorylation

RTK proximity promotes phosphorylation and activates downstream signaling proteins, influencing cancer cell behavior.^56^ EGFR, a key RTK, typically clusters on cancer cell membranes at distances ranging from approximately 70 to several hundred nanometers.^57^ Given the proximity-induced nature of EGFR phosphorylation,^42,58^ we employed high-affinity EGFR aptamers^52^ on DNA origami to precisely control ligand spacing and valency, enabling systematic investigation of EGFR activation. To this end, DNA origami structures were designed with interaptamer spacings ranging from 120 to 30 nm (see the AFM images in Figures 2g and S14a). Agarose gel analysis confirmed the successful functional assembly of the ligands with the DNA nanostructure (Figure S14c). Our results indicate that EGFR phosphorylation increases as the receptor spacing is reduced from 120 to 90 nm, with a 1.3-fold enhancement observed at 90 nm compared to the aptamer-free control (DO). However, further decreasing the spacing to 60 and 30 nm did not lead to additional increases in EGFR phosphorylation, likely due to insufficient aptamer density limiting effective EGFR clustering and activation (Figure 2h).

To further enhance EGFR phosphorylation, we increased aptamer density by incorporating adjacent EGFR aptamers at 30 nm spacing, where RTK phosphorylation is likely driven by proximity dimerization.^58^ By increasing the EGFR aptamer density from 58 ± 10 aptamers/μm^2^ (two aptamers per DNA origami, DO_2E) to 174 ± 32 aptamers/μm^2^ (six aptamers per DNA origami, DO_6E) (see the AFM images in Figures 2g and S14b and agarose gel analysis in Figure S14d), we observed a significant boost in phosphorylation. EGFR phosphorylation increased 1.7-fold at 174 ± 32 aptamers/μm^2^ compared to DO, while lower densities of 58 ± 11 and 116 ± 22 aptamers/μm^2^ yielded smaller increases of 1.3- and 1.5-fold, respectively (Figure 2i). These results show that EGFR activation depends on both spacing and cluster density, with high local concentrations required for robust phosphorylation.

To confirm that phosphorylation was induced by the proximity of EGFR aptamers, we treated A375P β6 cells with the EGFR inhibitor gefitinib.^59^ In A375P β6 cells on the DO_6E substrate, p-EGFR levels were significantly higher than those on aptamer-free substrates (DO). However, upon addition of gefitinib, signaling was reduced to levels similar to those of the control group (Figure 2j), confirming the role of aptamer proximity in activating cell signaling.

### Integrin αvβ6–RTK Crosstalk Drives Cancer Cell Spreading and Coactivates FAK and RTK Signaling

To systematically investigate the stoichiometric crosstalk between integrins and RTKs, we focused on EGFR, HER2, and Met, which are known to functionally coordinate with integrin αvβ6 to regulate key cancer processes such as adhesion, migration, invasion, and metastasis^8,60^ (Figure 3a). Building upon this background, we developed DNA origami with three peptide modifications spaced 120 nm apart [designated as DO_3PP (120 nm), hereafter referred to as DO_3PP]. Initial tests showed limited cell spreading (Figure 2a–d). Drawing from our previous finding that a 3:6 αvβ6/EGFR ligand ratio (equivalent to 1:2) optimally promotes melanoma cell spreading,^28^ we incorporated six RTK-specific aptamers (EGFR, HER2, and Met) to create three configurations: DO_3PP_6E (EGFR), DO_3PP_6H (HER2), and DO_3PP_6M (Met) (see the AFM images in Figures 3c and S15a and gel analysis in Figure S15b). The results demonstrate the synergistic modulation of A375P β6 cell spreading using DNA origami. On high-spacing peptide substrates (DO_3PP) and RTK aptamer-only substrates (DO_6E, DO_6H, and DO_6M), only a few cells with minimal spreading were observed. However, combining peptides with RTK aptamer modifications (DO_3PP_6E, DO_3PP_6H, and DO_3PP_6M) significantly enhanced cell attachment and showed fully spread morphologies (Figure 3d).

**Figure 3:**
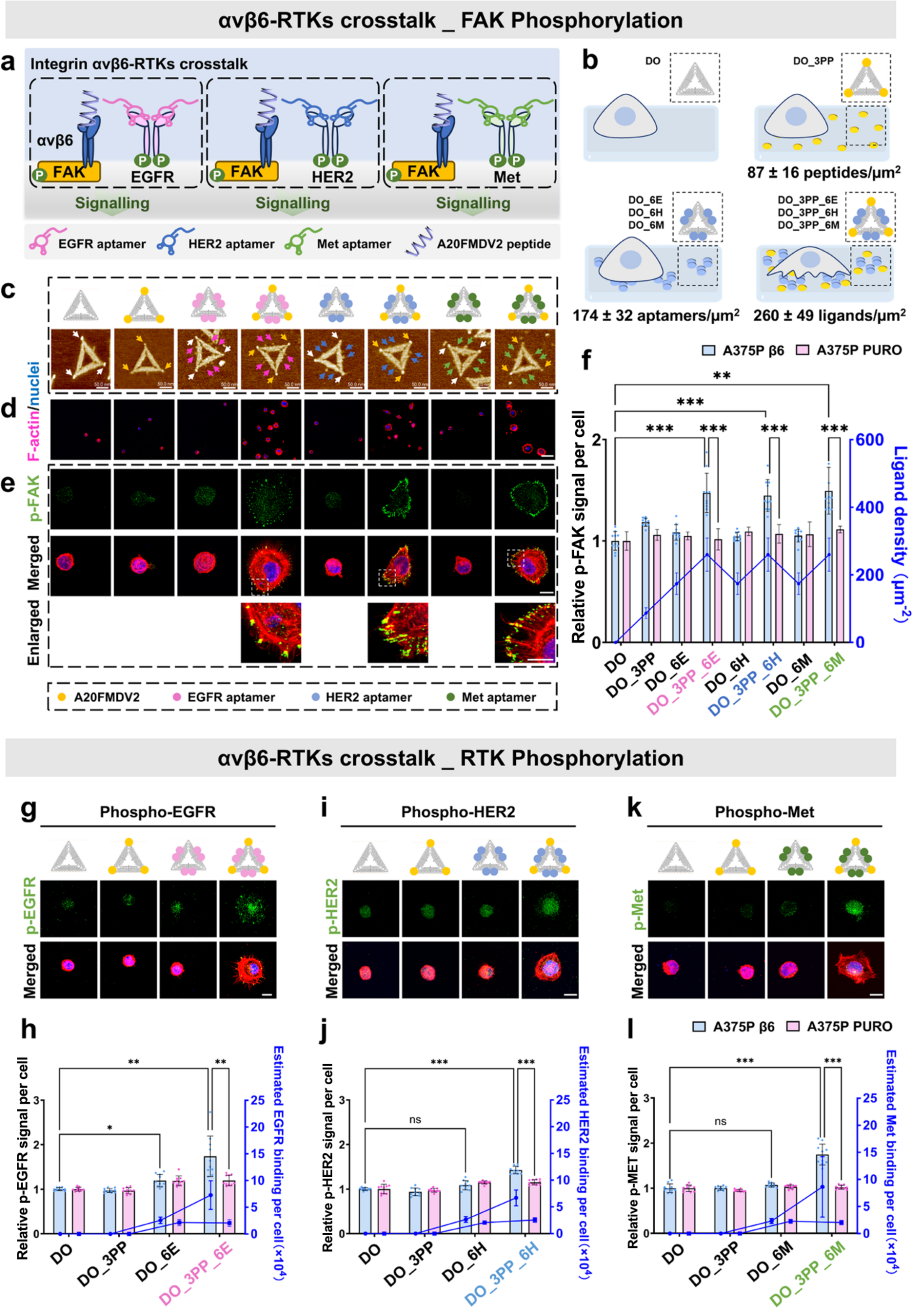
Spatially patterned A20FMDV2 and RTK aptamers synergistically enhance cell spreading and activate FAK and RTK phosphorylation. (a) Scheme of DNA origami-directed nanoarrangement of ligand regulating αvβ6–RTK (EGFR, HER2, and Met) crosstalk. (b) Illustration of A375P β6 cell spreading regulated by DNA origami-directed ligand copresentation. (c) AFM images of peptide- and aptamer-functionalized DNA origami. White arrow, streptavidin; yellow arrow, streptavidin-peptide; pink arrow, EGFR aptamer; blue arrow, HER2 aptamer; green arrow, Met aptamer. (d) Confocal images of the A375P β6 cell spreading on peptide- and aptamer-functionalized substrates. F-actin, red; nuclei, blue. Scale bar, 40 μm. Additional representative single-cell morphologies and quantitative analyses of cell spreading are shown in Figures S16–S18. (e) Representative Z-projected confocal image of p-FAK (Y397, green) in A375P β6 cells spreading on peptide- and aptamer-functionalized substrates. Scale bar, 10 μm. Enlarged image scale bar: 20 μm. Additional large-field overviews are provided in Figure S19. (f) Left *Y*-axis: normalized p-FAK intensity in A375P β6 (blue) and puro (pink) cells, with DO as the control (two-way ANOVA, *n* = 10, **P < 0.01, ***P < 0.001). Right *Y*-axis: corresponding ligand densities (peptides and RTK aptamers) of substrates. Data represent mean ± SD. (g, i, k) Representative Z-projected confocal images of (g) phospho-EGFR, (i) phospho-HER2, and (k) phospho-Met staining (green) in A375P β6. Scale bar, 10 μm. Additional large-field overviews are provided in Figure S20. (h, j, l) Left *Y*-axis: normalized (h) phospho-EGFR, (j) phospho-HER2, and (l) phospho-Met fluorescence intensity in A375P β6 and A375P puro cells, with DO as the control (two-way ANOVA, *n* = 10, *P < 0.05, **P < 0.01, ***P < 0.001; ns, not significant). Right *Y*-axis: estimated aptamers binding per cell calculated from the aptamer density and projected cell area. Data represent mean ± SD.

To quantitatively analyze cell spreading, the average spreading area was calculated and correlated with ligand density based on 20 single cells (Figure S16 for A375P β6 cells; Figure S17 for A375P puro cells). At the optimized EGFR aptamer density (174 ± 32 aptamers/μm^2^), p-EGFR signaling was detectable, but it failed to promote significant cell spreading. Similarly, a peptide density of 87 ± 16/μm^2^ provided insufficient mechanical support for robust focal adhesion formation and subsequent cell spreading. Notably, when A20FMDV2 peptides and EGFR aptamers were combined at identical densities (87 ± 16 peptides/μm^2^ and 174 ± 32 aptamers/μm^2^) with a 1:2 ratio (DO_3PP_6E), the single A375P β6 cell attachment area increased by 3.3-fold compared to DO. This cooperative effect was consistently observed in αvβ6–HER2 (DO_3PP_6H, 3-fold) and αvβ6–Met (DO_3PP_6M, 3.9-fold) crosstalk, demonstrating the generalizability of this synergistic mechanism (Figure S18).

During the attachment process, αvβ6–EGFR, αvβ6–HER2, and αvβ6–Met crosstalk demonstrated significant FA formation and colocalization at protrusive structures (Figure 3e), with p-FAK increases of 1.5-, 1.4-, and 1.5-fold, respectively, compared to DO. These enhancements notably exceeded the 1.2-fold enhancement, which was observed in the 3PP_DO condition (Figure 3f). Representative Z-projected confocal images of p-FAK are shown in Figure 3e, while large-field overviews are presented in Figure S19 to illustrate the population-level consistency. These results demonstrated that copresentation of both ligands at optimized densities induced robust cell spreading, forming clusters that stabilized nascent adhesions and enabled FAK phosphorylation for spreading^61^ (Figure 3b). In contrast, A375P puro cells, lacking β6 integrin, failed to exhibit this synergistic effect (Figures 3f and S23a), highlighting the essential role of αvβ6 integrin in mediating these cooperative interactions.

Incorporating RTK-specific aptamers into the substrate significantly strengthens integrin-mediated cell spreading, expanding the cell membrane–substrate contact interface and resulting in more RTK aptamer engagement with cell receptors. This resulted in enhanced phosphorylation of EGFR (Figure 3g,h), HER2 (Figure 3i,j), and Met (Figure 3k,l). On DO_3PP_6E substrates, A375P β6 cells exhibited a 2.9-fold increase in EGFR aptamer binding per cell compared to DO_6E substrates, accompanied by a 55% further increase in phosphorylated EGFR (Figure 3h). Substrates with only HER2 or Met aptamers (DO_6H and DO_6M) did not exhibit direct p-HER2 and p-Met signaling, but the integrated platforms (DO_3PP_6H and DO_3PP_6M) showed significant phosphorylation increases of 1.4- and 1.7-fold, respectively, compared with those of DO (Figure 3j,l). These findings demonstrate that αvβ6–RTK complexes stabilize FAs and promote cell spreading, thereby extending the interface between ligands and receptors and further enhancing RTK activation. Interestingly, the confocal images reveal that, unlike p-FAK, which localizes mainly at the juxtamembrane region, phosphorylated RTKs are also observed in the cytoplasm and nucleus. RTK–ligand binding typically induces dimerization and internalization via endosomal trafficking. Since our ligands are covalently anchored to DNA origami adhered to the extracellular surface, the intracellular RTK signal likely reflects active receptor trafficking and signaling (Figure 3g,i,k). In contrast, A375P puro cells did not exhibit collaborative spreading behavior due to the absence of αvβ6 and lacked the additional RTK activation (Figures 3h,j,l and S23b–d).

### Integrin αvβ6–RTK Crosstalk Synergistically Activates PI3K–AKT and Ras–MAPK Pathways

Integrin αvβ6 is involved in downstream signaling with RTKs, notably through the PI3K–AKT and Ras–MAPK pathways.^3^ Crosstalk between integrins and RTKs affects the activity, expression level, signaling, and trafficking. In A375P β6 cells, coactivation of FAK and RTKs activates the PI3K–AKT pathway, leading to PI3K phosphorylation and AKT activation, which promotes cell survival and growth (Figure 4a). Figure 4c shows quantitative analyses of Z-projected confocal images of p-AKT levels in A375P β6 and A375P puro cells. Representative single-cell images are shown in Figure 4b, and large-field overviews are provided in Figure S21 to illustrate population-level consistency. The results reveal a positive correlation between p-AKT levels and the phosphorylation of RTKs (EGFR/HER2/Met) and FAK across ligand-functionalized substrates (see p-EGFR/p-HER2/p-Met and p-FAK data in Figure 3). Combinatorial αvβ6–RTK coactivation substrates (DO_3PP_6E/H/M, with a ligand density of 260 ± 49 peptides and aptamers/μm^2^) exhibited significantly elevated p-AKT intensities, with 2.1-, 1.9-, and 1.8-fold increases in αvβ6–EGFR, αvβ6–HER2, and αvβ6–Met activation, respectively, compared to DO. These activation levels markedly surpassed those observed in single-ligand substrates (DO_3PP and DO_6E/H/M, with 87 ± 16 peptides/μm^2^ or 174 ± 32 aptamers/μm^2^, respectively), which showed modest AKT activation (∼1.3-fold across all configurations). The p-AKT signal from αvβ6–RTK coactivation exceeded the sum of individual ligand activations, with αvβ6–EGFR, αvβ6–HER2, and αvβ6–Met crosstalk exhibiting 41, 31, and 27% higher signaling intensity, respectively (Figure 4c). These results demonstrate that our biomimetic DNA origami platform enables dissection of RTK–integrin crosstalk signaling at the single-molecule level. They support previous findings highlighting that integrin–RTK interactions coordinate downstream signaling through concomitant activation of parallel pathways via receptor colocalization and through collaborative coupling within adhesion complexes.^10^

**Figure 4:**
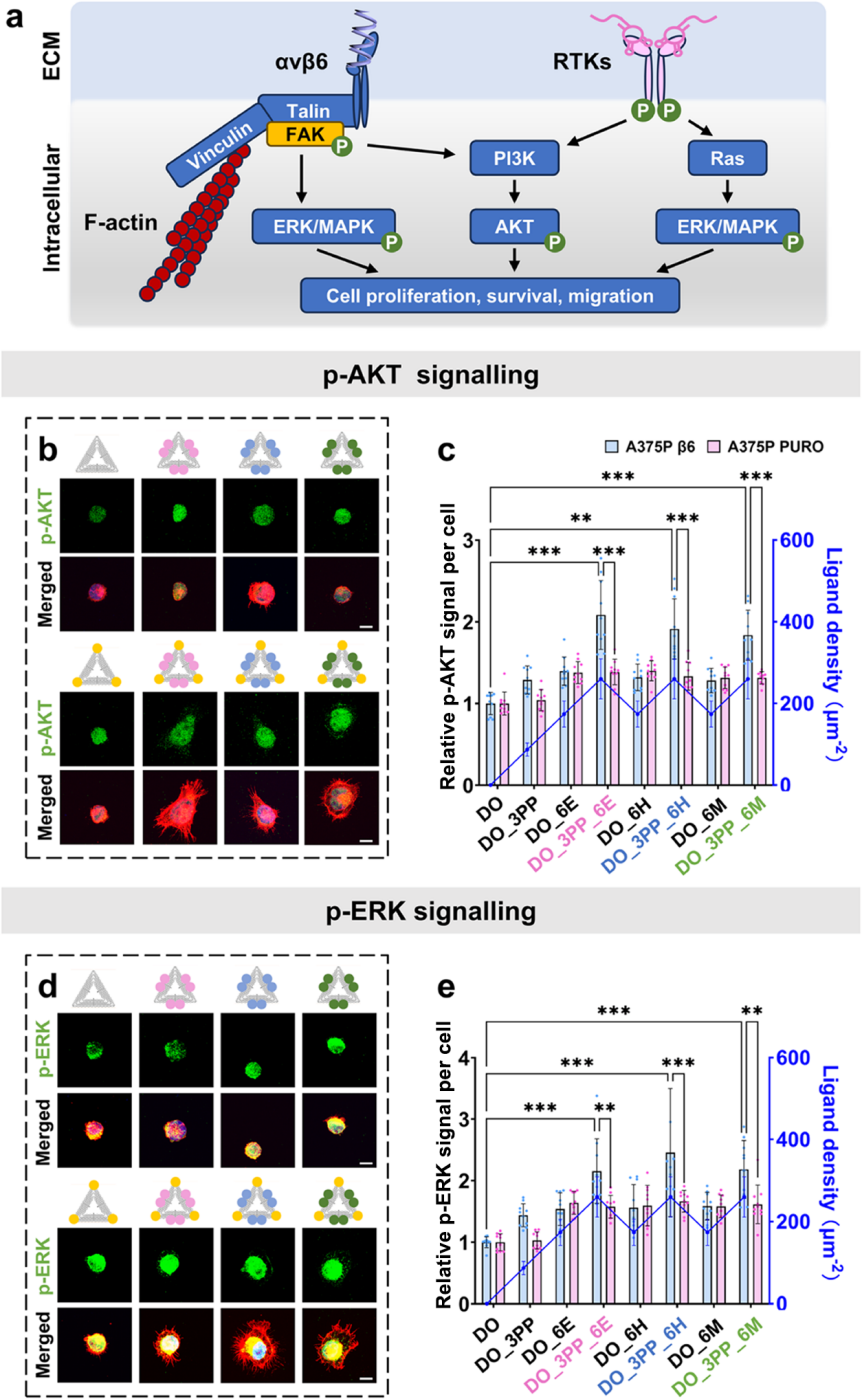
Spatially patterned A20FMDV2 and RTK aptamers synergistically activate the PI3K–AKT and Ras–MAPK pathways. (a) Schematic of PI3K–AKT and Ras–MAPK pathways in αvβ6–RTK crosstalk. (b) Representative Z-projected confocal images of p-AKT (green) in A375P β6 cells spreading on various functionalized substrates. F-actin, red; nuclei, blue. Scale bar, 10 μm. Additional large-field overviews are provided in Figure S21. (c) Left *Y*-axis: normalized p-AKT intensity in A375P β6 (blue) and puro (pink) cells, with DO as the control (two-way ANOVA, *n* = 10, **P < 0.01, ***P < 0.001). Right *Y*-axis: corresponding ligand densities (peptides and RTK aptamers) of substrates. Data represent mean ± SD (d) Representative Z-projected confocal images of p-ERK (green) in A375P β6 cells spreading on various functionalized substrates. F-actin, red; nuclei, blue. Scale bar, 10 μm. Additional large-field overviews are provided in Figure S22. (e) Left *Y*-axis: normalized p-ERK intensity in A375P β6 (blue) and puro (pink) cells, with DO as the control (two-way ANOVA, *n* = 10, **P < 0.01, ***P < 0.001). Right *Y*-axis: corresponding ligand densities (peptides and RTK aptamers) of substrates. Data represent mean ± SD.

A similar αvβ6–RTK interaction occurs in the Ras–MAPK pathway, where the FA complex recruits adaptor proteins to activate Ras, triggering the MAPK/ERK cascade.^3^ Activated ERK (p-ERK) ultimately translocates to the nucleus to regulate genes associated with proliferation and migration (Figure 4a). Figure 4d demonstrates pronounced nuclei localization of p-ERK in cells adhering to our DNA origami synergistic ligand platforms (DO_3PP_6E/H/M), and large-field overviews are provided in Figure S22. Quantitative analysis revealed 2.2-, 2.5-, and 2.2-fold increases in p-ERK signaling for DO_3PP_6E, DO_3PP_6H, and DO_3PP_6M, respectively, compared to DO. These coactivation platforms demonstrated synergistic enhancements of 17, 45, and 15% beyond the additive effects of individual RTK and integrin activation, respectively (Figure 4e). In contrast, when only single ligand types were presented on the substrates, such as three peptides spaced at 120 nm (DO_3PP), p-FAK activation was not observed, indicating limited capacity to trigger downstream signaling. Similarly, although RTK proximity (*e.g.*, EGFR) induced by aptamers can initiate receptor phosphorylation, simply linking two EGFRs via a DNA origami scaffold is insufficient to recruit and activate Ras and thus less effective in triggering MAPK/ERK signaling^62^ (Figure 4e). These trends in p-ERK enhancement on copresented ligand substrates closely correlate with upstream activation of p-FAK and p-RTKs (Figure 3), highlighting the dual regulation by mechanosensitive FAK signaling and RTK-mediated Ras–MAPK activation. These findings further support that the copresentation of both ligands on DNA origami enables multivalent, single-molecule coordination of two distinct receptors, thereby reflecting their cooperative behavior. In A375P puro, DNA origami substrates incorporating both peptides and RTK aptamers exhibit weaker p-AKT and p-ERK signals (Figure 4c,e). Without αvβ6 integrin, activation mainly results from individual RTK triggering, which is limited (Figure S23e,f), confirming the critical role of αvβ6 integrin in mediating cooperative signaling interactions and synergistic downstream effects with RTKs.

### Integrin αvβ6–RTK Crosstalk Drives Cell-Type-Specific FAK and RTK Phosphorylation in Breast Cancer Cells

The spreading behavior of epithelial-morphology breast cancer cells is closely linked to molecular crosstalk between αvβ6 and RTKs, potentially influenced by cell-specific RTK expression profiles. To explore how this interaction regulates cell-type-specific adhesion and signaling, we used our DNA origami-based platform with spatially controlled integrin αvβ6 and RTK ligands in αvβ6-positive breast cancer cell lines MDA-MB-468 (triple-negative breast cancer, high EGFR, HER2-negative) and BT-474 (HER2-positive invasive ductal carcinoma, moderate Met expression) (Figures S4–S8).

In MDA-MB-468 cells, the presence of αvβ6 integrin led to a consistent response across substrates with varying A20FMDV2 peptide densities. As the peptide density increased (from 87 ± 16 peptides/μm^2^ to 347 ± 65 peptides/μm^2^), cell spreading and p-FAK activation enhanced (Figure S24), similar to A375P β6 cells. Using the same DNA origami configurations as in A375P β6 (DO_3PP, 87 ± 16 peptides/μm^2^; DO_6E/H/M, 174 ± 32 EGFR/HER2/Met aptamers/μm^2^; DO_3PP_6E/H/M, 260 ± 49 ligands/μm^2^), we observed that combining A20FMDV2 peptides with EGFR/MET aptamers (DO_3PP_6E/6M) synergistically enhanced the cell spreading area (1.8-fold for DO_3PP_6E and 1.6-fold for DO_3PP_6M, respectively, *vs* DO; Figures 5a, S25, and S26) and elevated FAK phosphorylation (1.4- and 1.5-fold enhancement, respectively, *vs* DO) compared to peptide-only controls (DO_3PP, only 1.2-fold *vs* DO; Figure 5b,c). Due to the HER2-negative status of MDA-MB-468, DO_3PP_6H did not significantly promote cell spreading (Figure 5a) and induced only a modest 1.2-fold increase in p-FAK, consistent with DO_3PP activation (Figure 5b). In contrast, BT-474 cells exhibited significantly enhanced spreading area on all combinatorial substrates (DO_3PP_6E/6H/6M), with approximately 2-fold increases for DO_3PP_6E/6H and 1.8-fold for DO_3PP_6M, whereas single-ligand configurations showed no detectable changes (Figures 5d, S27, and S28). Despite lower β6 expression, all combinatorial groups showed a modest ∼1.3-fold increase in p-FAK, surpassing the response observed with individual ligands (Figure 5e,f).

**Figure 5:**
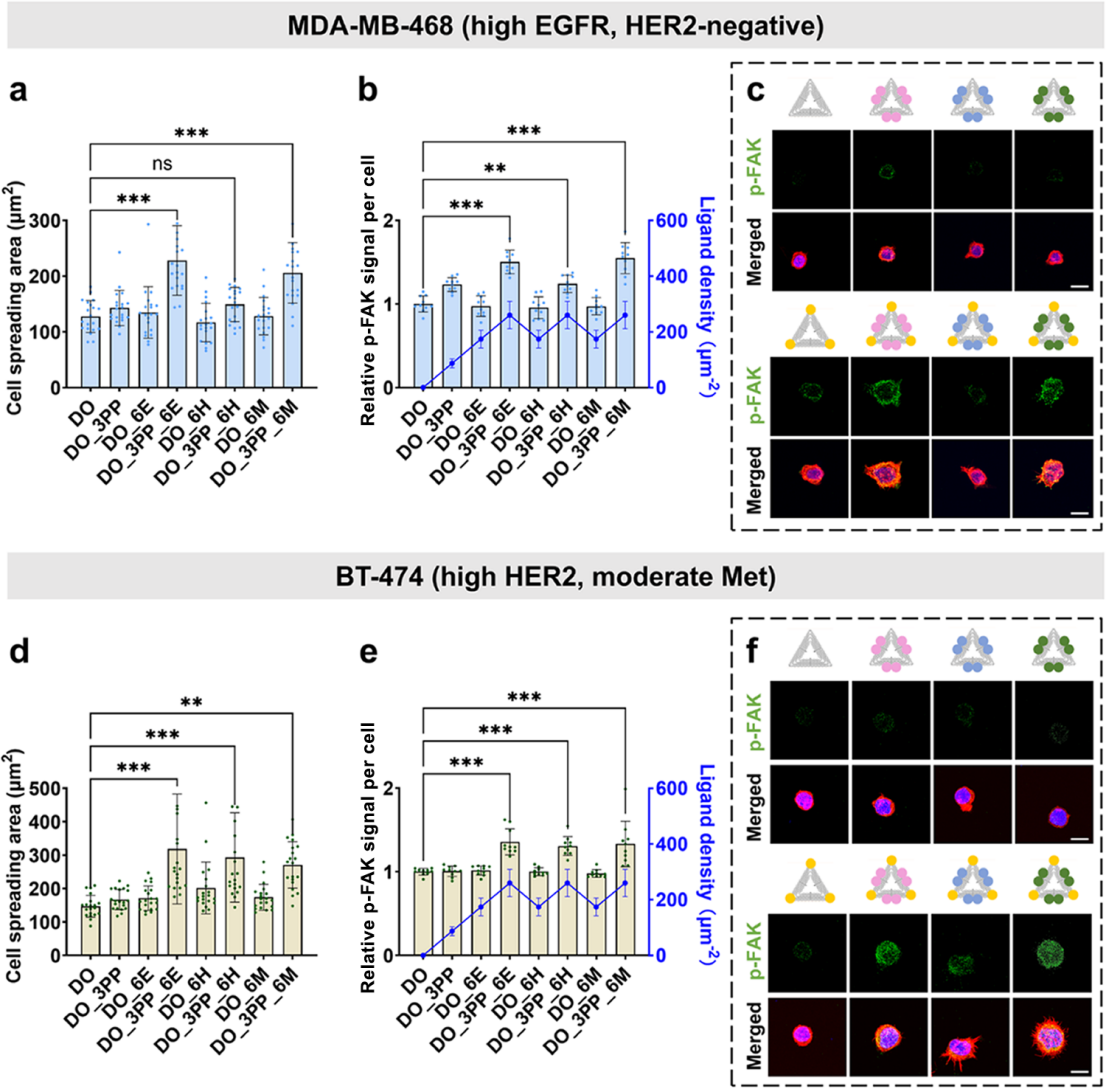
Spatially patterned A20FMDV2 and RTK aptamers drive cell-type-specific FAK activation in breast cancer cell models. (a, d) Single-cell quantitative morphometric analysis of (a) MDA-MB-468 and (d) BT-474, showing the impact of ligand crosstalk on cell spreading area, with DO as the control (one-way ANOVA, **P < 0.01, ***P < 0.001; ns, not significant). Data represent mean ± SD. Additional representative images used for quantification are shown in Figure S25 (MDA-MB-468) and Figure S27 (BT-474). (b, e) Left *Y*-axis: normalized p-FAK intensity in (b) MDA-MB-468 and (e) BT-474, with DO as the control (one-way ANOVA, *n* = 10, **P < 0.01, ***P < 0.001). Right *Y*-axis: corresponding ligand densities (peptides and RTK aptamers) of substrates. Data represent mean ± SD (c, f) Representative Z-projected confocal image of p-FAK (Y397, green) in (c) MDA-MB-468 and (f) BT-474 cells spreading on functionalized substrates. Scale bar,10 μm. F-actin, red; nuclei, blue.

Regarding RTK activation, MDA-MB-468 on DO_3PP_6E demonstrated a 2.5-fold increase in p-EGFR compared to a 1.5-fold increase in DO_6E (Figure 6a). Similarly, DO_3PP_6M exhibited a 2.1-fold increase in p-Met, while DO_6M showed undetectable p-Met levels (Figure 6c). Nevertheless, DO_3PP_6H and DO_6H showed no significant effect on p-HER2 (Figure 6b), consistent with the cells’ HER2-negative status. In BT-474 cells, RTK activation was significantly increased in all combinatorial groups, with a 1.5-fold increase in p-EGFR (Figure 6d) and a moderate 1.3-fold increase in p-Met (Figure 6f). Notably, HER2 aptamer substrates (DO_6H) alone induced a 1.5-fold increase in p-HER2, consistent with receptor overexpression in this cell line. However, combining them with integrin ligands (DO_3PP_6H) resulted in a slight additional increase (1.7-fold), reflecting limited β6 expression in BT-474 cells (Figure 6e). These results demonstrate the cell-type-specific nature of integrin–RTK crosstalk and its dependence on receptor expression profiles, highlighting the ability of DNA origami to dissect nanoscale signaling crosstalk across different cell types.

**Figure 6:**
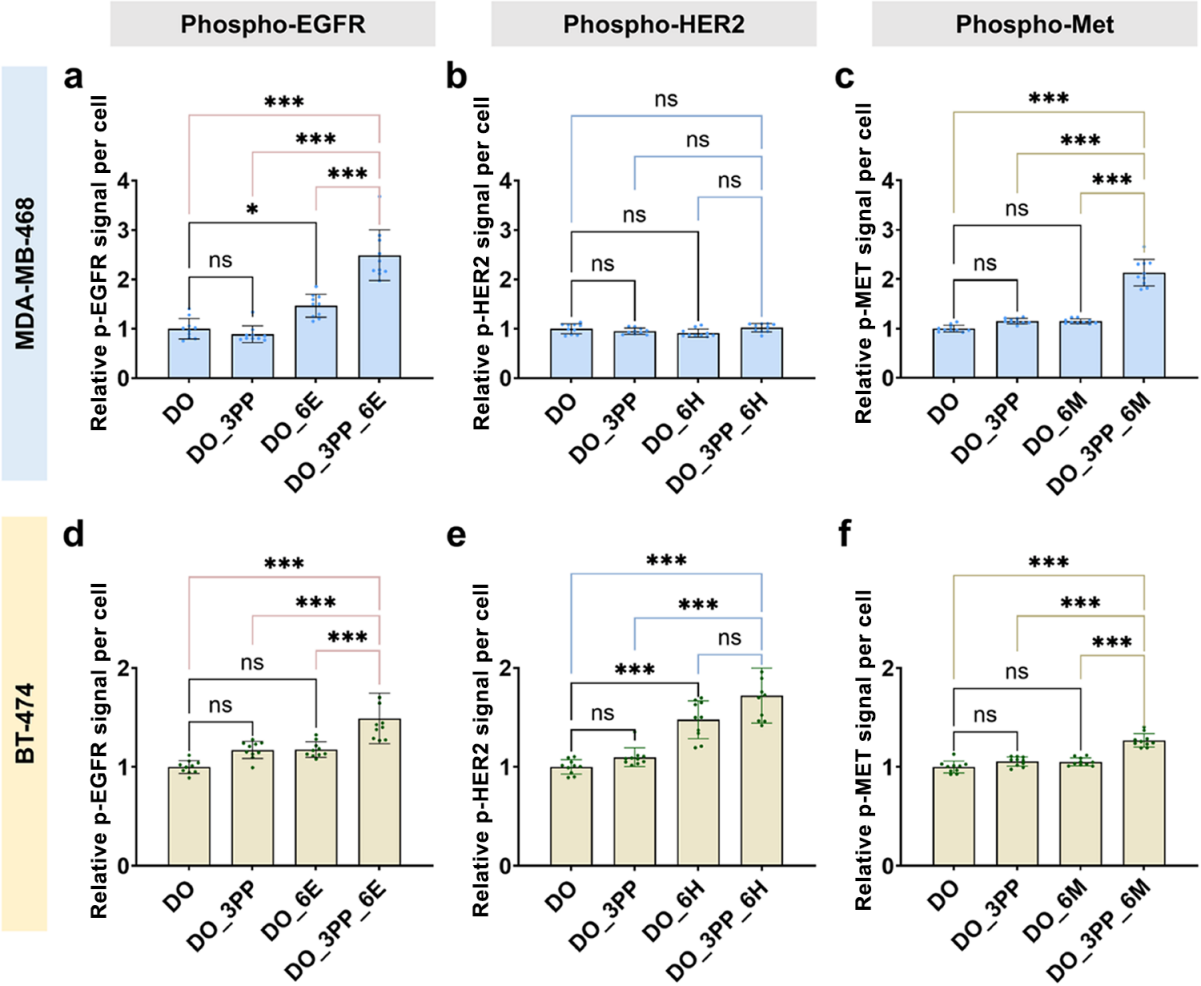
Spatially patterned A20FMDV2 and RTK aptamers drive cell-type-specific RTK (EGFR/HER2/Met) activation in breast cancer cell models. Fluorescence intensity of (a) normalized p-EGFR, (b) p-HER2, and (c) p-Met in MDA-MB-468 cells. Fluorescence intensity of (d) normalized p-EGFR, (e) p-HER2, and (f) p-Met in BT-474 cells. DO serves as a control (one-way ANOVA, *n* = 10, *P < 0.05, ***P < 0.001; ns, not significant). Data represent mean ± SD.

## Conclusions

In this study, we developed a DNA origami-based biomimetic platform to control ligand multivalency and spatial organization at the nanoscale, enabling the systematic investigation of αvβ6–RTK crosstalk in cancer biology. Due to the programmability of DNA origami, which allows precise control over ligand number and spatial distribution, we could rationally design ligand configurations that mimic the ECM, relevant to the aforementioned biological cooperative behavior. By observing cell behaviors and phosphorylation levels in response to defined nanoenvironments, we could determine the minimal ligand number and arrangement required to activate cellular signaling, thus demonstrating how intricate biological processes can be quantitatively dissected using a DNA nanotechnology strategy. Specifically, our results reveal three key design principles: (i) a spatial activation threshold for A375P β6 cell spreading, requiring αvβ6-specific peptide (A20FMDV2) densities of 87 ± 16 peptides/μm^2^ at ≤60 nm spacing; (ii) spacing- and density-dependent EGFR phosphorylation induced by EGFR aptamers; and (iii) at a ligand density of 260 ± 49 ligands/μm^2^ and an optimized peptide-to-RTK aptamer ratio of 1:2, αvβ6–RTK (EGFR, HER2, and Met) coactivation synergistically enhanced cancer cell spreading and amplified downstream PI3K–AKT and Ras–MAPK/ERK signaling, exceeding the additive effects of individual activation. Validation in breast cancer models (MDA-MB-468 and BT-474) showed cell-type-specific integrin–RTK crosstalk depending on RTK and integrin expression levels. This platform provides mechanistic insights into how spatially organized integrin αvβ6 engages in crosstalk with RTKs to regulate downstream signaling, offering a framework for developing metastatic microenvironment mimics and therapeutic strategies. Our findings highlight the critical role of nanoscale ligand patterning and multivalency in enhancing cancer cell behavior, potentially advancing precision-targeted interventions in integrin–RTK-driven malignancies.

## Methods

### DNA Origami Synthesis, Purification, and Characterization

Triangular DNA origami structures^23^ were assembled by mixing 10 nM single-stranded M13mp18 scaffold DNA with 100 nM staple strands (sequences provided in Supporting Tables) in 50 μL of 1× assembly buffer (1× TAE with 12.5 mM MgCl_2_). The mixture was annealed from 95 to 20 °C using a thermal cycler at a cooling rate of 1 °C/min and stored at 4 °C after annealing. Self-assembled DNA origami was purified using Millipore Amicon Ultra 100 kDa spin columns at 10,000 rpm for 4 min, and the process was repeated three times to remove excess staple strands. A Nanodrop spectrophotometer was used to detect the concentrations of DNA origami products. Subsequently, agarose gel electrophoresis and atomic force microscopy (AFM) analysis were performed to analyze the DNA origami assembly.

### Conjugation of A20FMDV2 Peptides and RTK Aptamers to DNA Origami

A biotin–streptavidin binding strategy was employed to conjugate A20FMDV2 peptides to DNA origami. First, 16-nucleotide single-stranded DNA (ssDNA) with a 5′ biotin modification, complementary to a predefined sticky end on the DNA origami, was hybridized to introduce biotin handles for peptide attachment. Streptavidin was then added at a tenfold molar excess relative to the number of biotinylated sites on the DNA origami (maintained at 2 nM or lower to prevent aggregation) and incubated for 30 min. Subsequently, biotinylated A20FMDV2 peptides were added at a tenfold molar excess relative to streptavidin and incubated for an additional 30 min, ensuring that each streptavidin molecule could simultaneously bind to the peptides.

Additionally, site-specific modifications of RTK aptamers (EGFR, HER2, and Met) were achieved by hybridizing complementary sequences with the corresponding sticky ends on the DNA origami. This process was carried out overnight at 4 °C. Successful conjugation of both A20FMDV2 peptides and aptamers was confirmed by AFM imaging and agarose gel electrophoresis.

### AFM Characterization of DNA Origami

DNA origami structures were characterized using a Dimension Icon AFM (Bruker). For optimal dispersion, the DNA origami solution was diluted to ∼1 nM using 1× assembly buffer. A 5 μL aliquot was deposited onto a freshly cleaned mica surface and incubated for 5 min to allow surface adsorption. The substrate was then rinsed with Milli-Q water to remove unbound structures and dried by using compressed air. Imaging was performed in ScanAsyst mode using ScanAsyst-Air probes with a resolution of 512 pixels/line and a scan rate of 1 Hz.

### Agarose Gel Electrophoresis

A 2% agarose gel was prepared in a 1× TAE buffer containing 12.5 mM MgCl_2_. DNA origami samples were mixed with loading dye and loaded into wells. Electrophoresis was conducted at 70 V for 120 min to separate the DNA structures. Following electrophoresis, the gel was stained with SYBR Gold and imaged using an iBright FL1500 system (Thermo Fisher Scientific).

### DNA Origami Covalent Immobilization

This method was performed as previously described.^63,64^ To enable covalent immobilization of DNA origami onto substrate surfaces, 15 amino groups were introduced along the inner edges of triangular DNA origami structures (sequences provided in Supporting Tables). The DNA origami was diluted to 300 pM in 5 mM Tris buffer containing 35 mM MgCl_2_ (pH 8.3), and 100 μL of the solution was deposited onto a 13 mm coverslip that had been treated with oxygen plasma for 30 min prior to use. The samples were placed on a 24-well plate with a moist Kimwipe and incubated on a shaker for 90 min. Following adsorption, the substrate was washed three times with the same buffer. A 0.01% carboxyethylsilane solution (100 μL) in the same buffer was then added and incubated for 2 min with gentle shaking. The buffer was exchanged with 10 mM MOPS buffer with 125 mM MgCl_2_ (pH 8.1), and the substrate was washed three times. For covalent conjugation, a freshly prepared solution of 50 mM EDC and 100 mM NHS in the MOPS buffer was added and incubated for 10 min. After reaction, the substrate was washed with 10 mM MOPS containing 150 mM NaCl (pH 8.1), followed by rinsing with DPBS supplemented with 125 mM NaCl to remove noncovalently bound DNA origami. The functionalized substrates were stored in DPBS until further use.

### Cell Culture

Human melanoma cell lines (A375P puro and A375P β6) were maintained in Dulbecco’s modified Eagle’s medium (DMEM) supplemented with 10% fetal bovine serum (FBS). The human triple-negative breast cancer cell line MDA-MB-468 and invasive ductal carcinoma cell line BT-474 were cultured in RPMI-1640 medium with 10% FBS. All cells were incubated at 37 °C in a humidified atmosphere of 5% CO_2_. Cells were passaged approximately every 3 days using 0.25% (w/v) trypsin–EDTA for detachment, followed by neutralization with fresh culture medium and reseeding into new tissue culture flasks.

### Cell Spreading Assay

Cells were detached from tissue culture flasks using trypsin, neutralized with fresh medium, pelleted by centrifugation, and resuspended as previously described. Cell concentrations for both lines were determined by using a cell counting chamber. Equal numbers of cells (3 × 10^4^) were seeded onto functionalized substrates placed in 24-well plates. Before cell seeding, substrates were blocked with 1% BSA for 1 h. After 1.5 h of incubation, nonadherent cells were removed by gentle PBS washing. Cells were fixed in 4% formalin for 10 min at room temperature, followed by PBS rinsing. Cell morphology was assessed by microscopy, and cell number, projected area, and perimeter were quantified using ImageJ.

### Inhibition of EGFR Phosphorylation

Gefitinib, a potent and selective EGFR inhibitor, was dissolved in 100% DMSO at a stock concentration of 10 mg/mL and then diluted 1000-fold in culture medium, and the final concentration of DMSO in the culture medium did not exceed 0.1% (v/v). Cells were pretreated with gefitinib at 10 μg/mL for 15 min prior to seeding on DNA origami-functionalized substrates to inhibit EGFR phosphorylation.

### Immunofluorescent Staining for Analysis of Cancer Cell Signaling

Following fixation, cells were permeabilized with 0.1% Triton X-100 in PBS for 10 min at room temperature and washed three times with flow buffer. Samples were then incubated with primary antibodies (detailed in Supporting Tables) overnight at 4 °C. After three additional washes with flow buffer, Alexa Fluor 488-conjugated secondary antibodies (1:200 dilution in flow buffer) were applied for 30 min at room temperature. Rhodamine-phalloidin and DAPI were subsequently added and incubated for 10 min at room temperature in the dark. After two final washes with flow buffer and one rinse with Milli-Q water, samples were mounted onto glass slides by using ProLong Gold Antifade Mountant. Imaging was conducted using a Leica Stellaris 8 confocal microscope equipped with a 63× oil objective.

### Cell Adhesion Quantification

Cells were seeded on differently functionalized substrates for 1.5 h and then fixed and stained under identical conditions. All quantitative experiments were performed with at least three independent biological replicates, which showed consistent trends and uniform fluorescence signals. (i) Adherent cell count: Adherent cells within a 300 μm × 300 μm field of view were counted to assess adhesion efficiency. Five fields were analyzed per condition. (ii) Single-cell morphological analysis (spreading area and perimeter): F-actin-stained cells were analyzed using threshold-based segmentation and particle analysis in ImageJ. Each cell was imaged within consistently sized regions of interest (ROIs) using 40-layer Z-stacks, and the maximum projected area was recorded. For each condition, 10–20 cells were analyzed per replicate. (iii) Phosphorylation signal quantification: Single-cell fluorescence intensity was calculated as the sum across 40 Z-stack slices covering the full cell height. Ten cells were selected from at least three different fields of view per experiment. ROIs of uniform sizes were used to ensure that each contained a single cell. Fluorescence intensities were measured in LAS X instrumentation using consistent threshold settings. For each condition, signals were normalized to the control sample (DNA origami without ligands, DO). Representative wide-field images demonstrating reproducibility and uniform fluorescence are provided in Supporting Figures.

### Flow Cytometry

Cell surface expression of the αvβ6 integrin was quantified using flow cytometry. Briefly, cells were harvested and resuspended in flow buffer (DMEM supplemented with 0.1% BSA and 0.1% sodium azide) to generate single-cell suspensions. Aliquots containing 1 × 10^4^ cells were incubated for 30 min at 37 °C with either αvβ6-specific primary antibody (10D5, 1:10 dilution) or a mouse IgG isotype control (1:250 dilution). After three washes with flow buffer, cells were incubated on ice for 30 min with Alexa Fluor 488-conjugated donkey antimouse IgG secondary antibody (Jackson ImmunoResearch, 1:200 dilution). Cells were then washed three more times, centrifuged, and resuspended in 1 mL of flow buffer containing DAPI (1:10,000 dilution) for live/dead discrimination. Flow cytometry was performed using a BD LSRFortessa cell analyzer, and the data were analyzed with FlowJo software.

### Western Blot Analysis

For protein analysis, cells were lysed in RIPA buffer and centrifuged at 13,000×*g* for 10 min at 4 °C. Protein concentrations were quantified using a BCA assay. Equal amounts of protein (30 μg per lane) were resolved on 4–12% bis-Tris SDS-PAGE gels at 80 V for 30 min, followed by 120 V for 1 h. Subsequently, proteins were transferred to nitrocellulose membranes at 150 V for 90 min.

Membranes were blocked with 5% BSA for 30 min and then incubated overnight at 4 °C with primary antibodies (1:1000, detailed in Supporting Tables), followed by incubation with species-specific secondary antibodies (1:2000, 1 h at room temperature). Signal detection was performed using enhanced chemiluminescence (ECL), and band intensities were quantified using ImageJ software.

### Statistical Analysis

Data are presented as mean ± standard deviation (SD). Statistical significance of single-cell morphometric and signaling intensity comparisons between A375P β6 and A375P puro cells was assessed using two-way analysis of variance (ANOVA), followed by Tukey’s posthoc test for multiple comparisons. For the analysis involving a single cell type seeded on different functionalized DNA origami substrates, one-way ANOVA with Tukey’s posthoc test was used. *P* values are indicated as follows: *P < 0.05, **P < 0.01, ***P < 0.001, and ns indicates not significant. All statistical analyses were performed using GraphPad Prism (version 10.4.0).

## Supplementary Material



## Notes

DNA origami design and characterization; stability assessment; β6 and RTK expression analysis by western blotting, flow cytometry, and immunofluorescence; quantitative morphometric analysis of cell spreading; phosphorylation signaling in A375P puro cells; and supporting tables providing detailed information on DNA sequences and antibodies used (PDF)

## Abbreviations

ECM: extracellular matrix
RTKs: receptor tyrosine kinases
EGFR: epidermal growth factor receptor
HER2: human epidermal growth factor receptor 2
Met: mesenchymal–epithelial transition factor
FAK: focal adhesion kinase
FAs: focal adhesions
AFM: atomic force microscopy
DO: DNA origami
