# Supplementary material for: Multivalent DNA Origami Enables Single-Molecule Dissection of Integrin αvβ6–Receptor Tyrosine Kinase Crosstalk in Cancer Biology
Source: ACS Nano. 2025 Aug 26;19(35):31467–80. doi: 10.1021/acsnano.5c07581 (PMC12424295; doi:10.1021/acsnano.5c07581)
Supplement: Supplementary file 1 [file nn5c07581_si_001.pdf]

Supporting Information

Multivalent DNA Origami Enables Single-Molecule Dissection of Integrin αvβ6-Receptor Tyrosine Kinase Crosstalk in Cancer Biology

Tingting Zheng[a], *Lauren Grace Rigby*[b], *John F. Marshall,*[b] * *Matteo Palma*[a] *

[a] Department of Chemistry, Queen Mary University of London, Mile End Road, London E1 4NS, United Kingdom, m.palma@qmul.ac.uk

[b] Barts Cancer Institute, Cancer Research UK Centre of Excellence, Queen Mary University of London, Charterhouse Square, London EC1M 6BQ, United Kingdom, j.f.marshall@qmul.ac.uk

Table of Contents

Supporting Figures **4**

Figure S1. Design and characterization of DNA origami. 4

Figure S2. Validation of DNA origami covalent immobilization. 5

Figure S3. Stability analysis of DNA origami. 6

Figure S4. Characterization of integrin β6 and RTK expression 7

Figure S5. Immunofluorescence analysis of β6 expression 8

Figure S6. Immunofluorescence analysis of EGFR expression 9

Figure S7. Immunofluorescence analysis of HER2 expression 10

Figure S8. Immunofluorescence analysis of Met expression 11

Figure S9. Density of DNA origami immobilized on the coverslip. 11

Figure S10. AFM and gel analysis of A20FMDV2 peptides functionalized DNA origami 12

Figure S11. Z-projected confocal images of A375P β6 cells on peptides-functionalized substrates. 13

Figure S12. Quantitative morphometric analysis of A375P β6 cells spreading on peptides-functionalized substrates. 14

Figure S13. Larger field-of-view images of p-FAK in A375P β6 cells on peptides-functionalized substrates. 14

Figure S14. AFM and gel analysis of EGFR aptamers-functionalized DNA origami. 15

Figure S15. AFM and gel analysis of hetero-ligand functionalized DNA origami. 16

Figure S16. Z-projected confocal images of A375P β6 cells on hetero-ligand functionalized substrates. 17

Figure S17. Z-projected confocal images of A375P puro cells on hetero-ligand functionalized substrates. 18

Figure S18. Single-cell quantitative morphometric analysis of A375P β6 and puro cells 19

Figure S19. Larger field-of-view Z-projected confocal images of p-FAK in A375P β6 cells on hetero-ligand functionalized substrates. 19

Figure S20. Larger field-of-view Z-projected confocal images of p-EGFR/p-HER2/p-Met in A375P β6 cells on hetero-ligand functionalized substrates. 20

Figure S21. Larger field-of-view Z-projected confocal images of p-AKT in A375P β6 cells on hetero-ligand functionalized substrates. 20

Figure S22. Larger field-of-view Z-projected confocal images of p-ERK in A375P β6 cells on hetero-ligand functionalized substrates. 20

Figure S23. Phosphorylation signaling in A375P puro cells on functionalized substrates. 21

Figure S24. Quantitative morphometric analysis of MDA-MB-468 cells spreading on peptides functionalized substrates. 22

Figure S25. Z-projected confocal images of MDA-MB-468 cells on hetero-ligand functionalized substrates. 23

Figure S26. Quantitative morphometric analysis of MDA-MB-468 on hetero-ligand functionalized substrates 24

Figure S27. Z-projected confocal images of BT-474 cells on hetero-ligand functionalized substrates. 25

Figure S28. Quantitative morphometric analysis of BT-474 on hetero-ligand functionalized substrates 26

Supporting Tables 27

Table S1. Sequences of amino anchors 27

Table S2. Sequences for EGFR aptamer patterning 28

Table S3. Sequences for A20FMDV2 peptide patterning 29

Table S4. Sequences of A20FMDV2 peptide 29

Table S5. Sequences for A20FMDV2 peptide and RTK aptamer co-patterning 30

Table S6. List of unmodified staple strands 31

Table S7. Immunofluorescence antibodies 38

Table S8. Immunoblotting antibodies 39

References 40

Supporting Figures

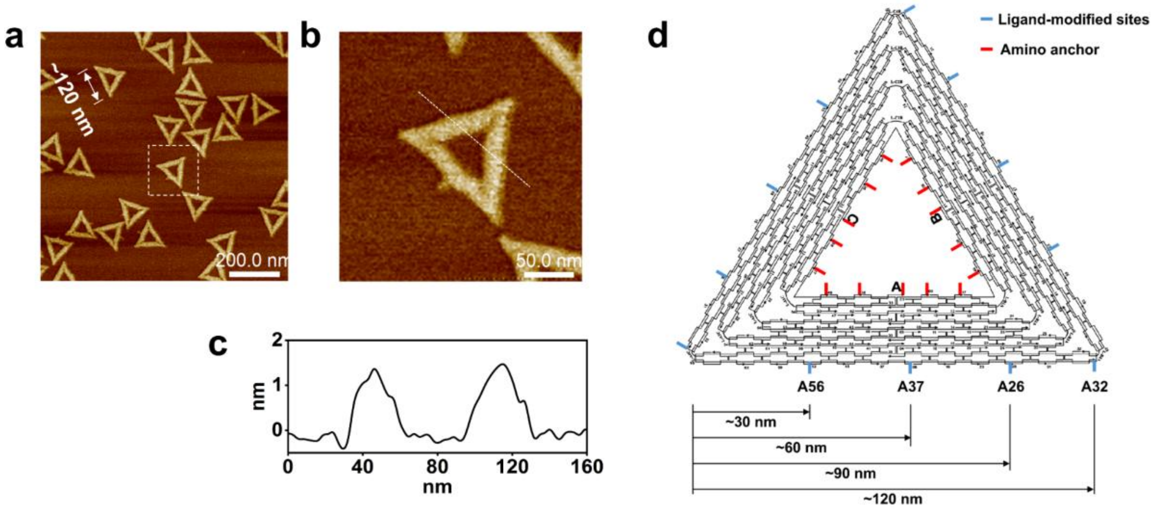

Figure S1. Design and characterization of DNA origami. (a) AFM image of triangular DNA origami. (b) Zoom-in of a single origami from (a). (c) Cross-sectional height profile of the structure shown in the inset of (b). (d) Schematic illustration showing 15 amino anchor sites and ligand (peptide/aptamer) modification positions on the triangular DNA origami.

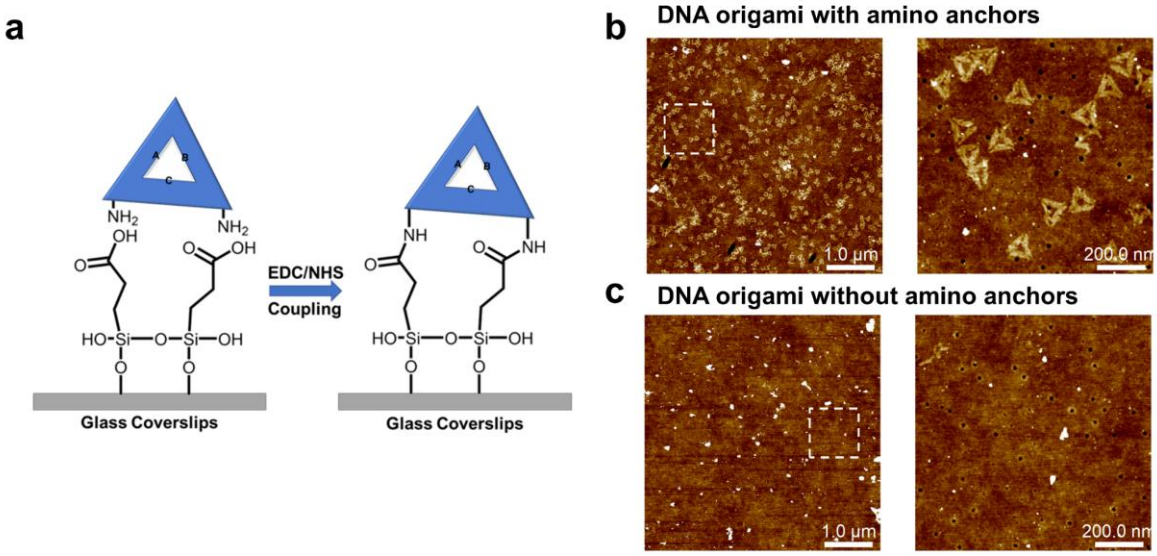

Figure S2. Validation of DNA origami covalent immobilization. (a) Reactions scheme of carboxylic silanization substrate used to couple with amino anchors of the DNA origami. (b) AFM image of DNA origami immobilized on the carboxylic silanized substrate via amino anchors; the structures remained surface-bound after rinsing. (c) AFM image of the DNA origami adsorbed onto the substrate without amino anchors, showing removal after rinsing.

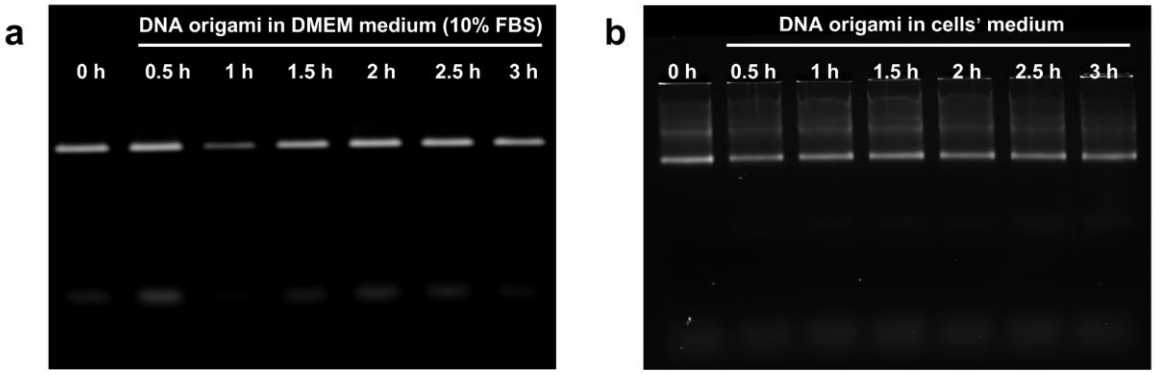

Figure S3. Stability analysis of DNA origami. Agarose gel electrophoresis analysis of DNA origami stability in (a) DMEM containing 10% FBS and (b) cell-conditioned medium (DMEM + 10% FBS, incubated overnight with A375P β6 cells), assessed at various time points (0, 0.5, 1, 1.5, 2, 2.5, and 3 hours) at room temperature.

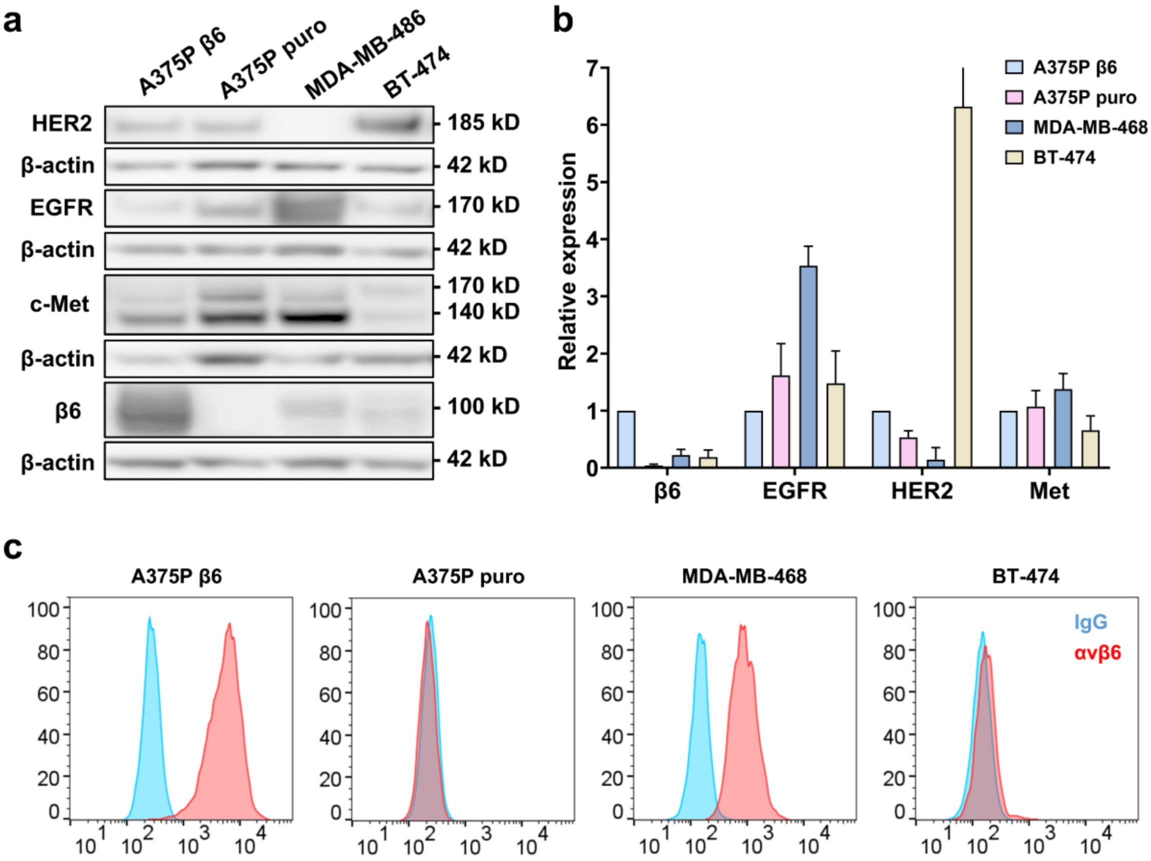

Figure S4. Characterization of integrin β6 and Receptor Tyrosine Kinase (RTK) expression in A375P β6, A375P puro, MDA-MB-468, and BT-474 cells. (a, b) Western blot analysis of integrin β6 and RTKs expression in A375P β6, A375P puro, MDA-MB-468, and BT-474 cells. (c) Flow cytometry analysis of surface β6 expression; IgG was used as an isotype negative control.

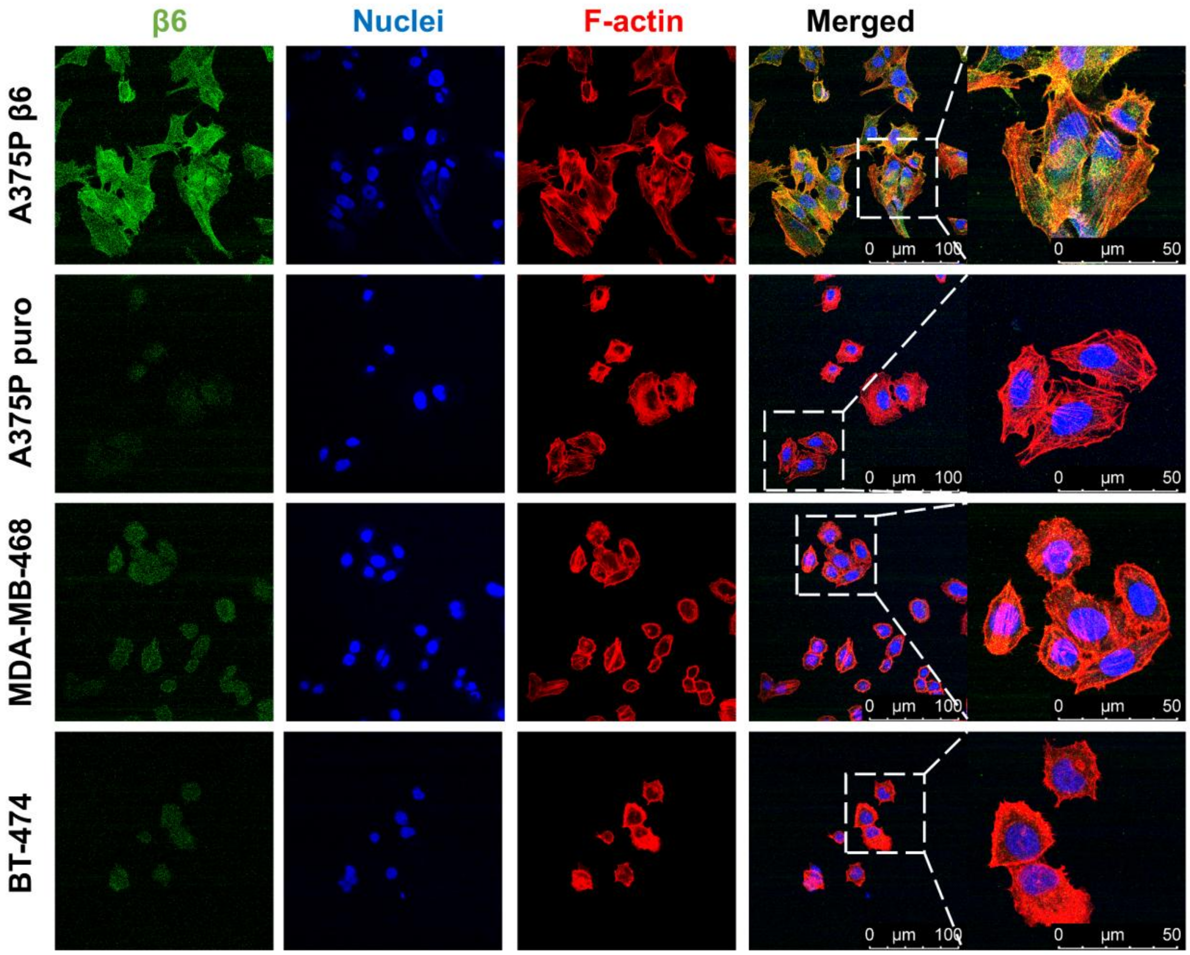

Figure S5. Immunofluorescence analysis of β6 expression in A375P β6, A375P puro, MDA-MB-468, and BT-474 cells. Cells were seeded on clean coverslips and allowed stable adhere for 24 hours before fixation and staining. Immunostaining with 10D5 antibody was used to assess β6 expression. All immunofluorescence data in **Figures S5-S8** were acquired under stable adhesion conditions. Single z slice shown at the juxtamembrane region. Scale bar, 50 μm. Green (β6); nuclei (blue); F-actin (red).

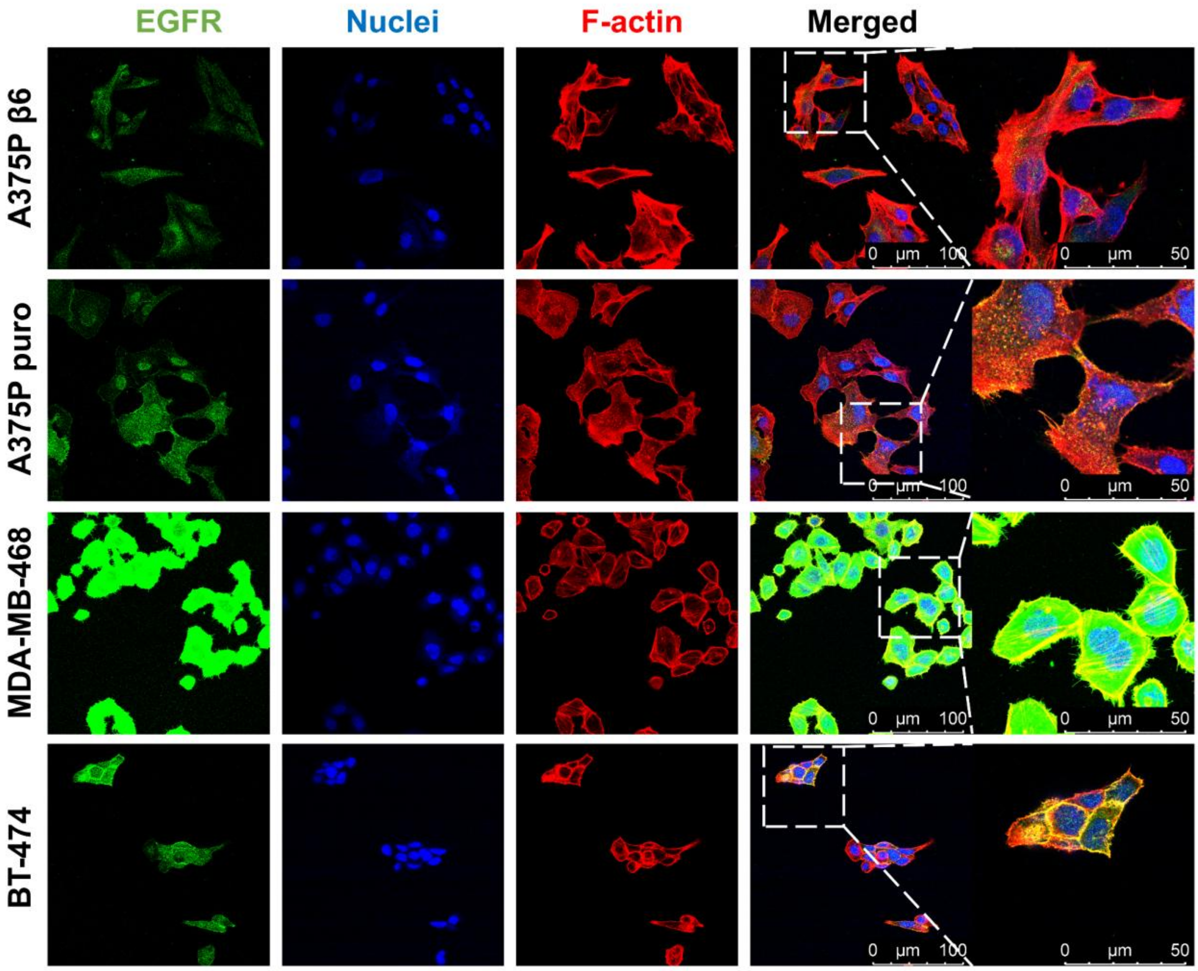

Figure S6. Immunofluorescence analysis of EGFR expression in A375P β6, A375P puro, MDA-MB-468, and BT-474 cells. Single z slice shown at the juxtamembrane region. Scale bar, 50 μm. Green (EGFR); nuclei (blue); F-actin (red).

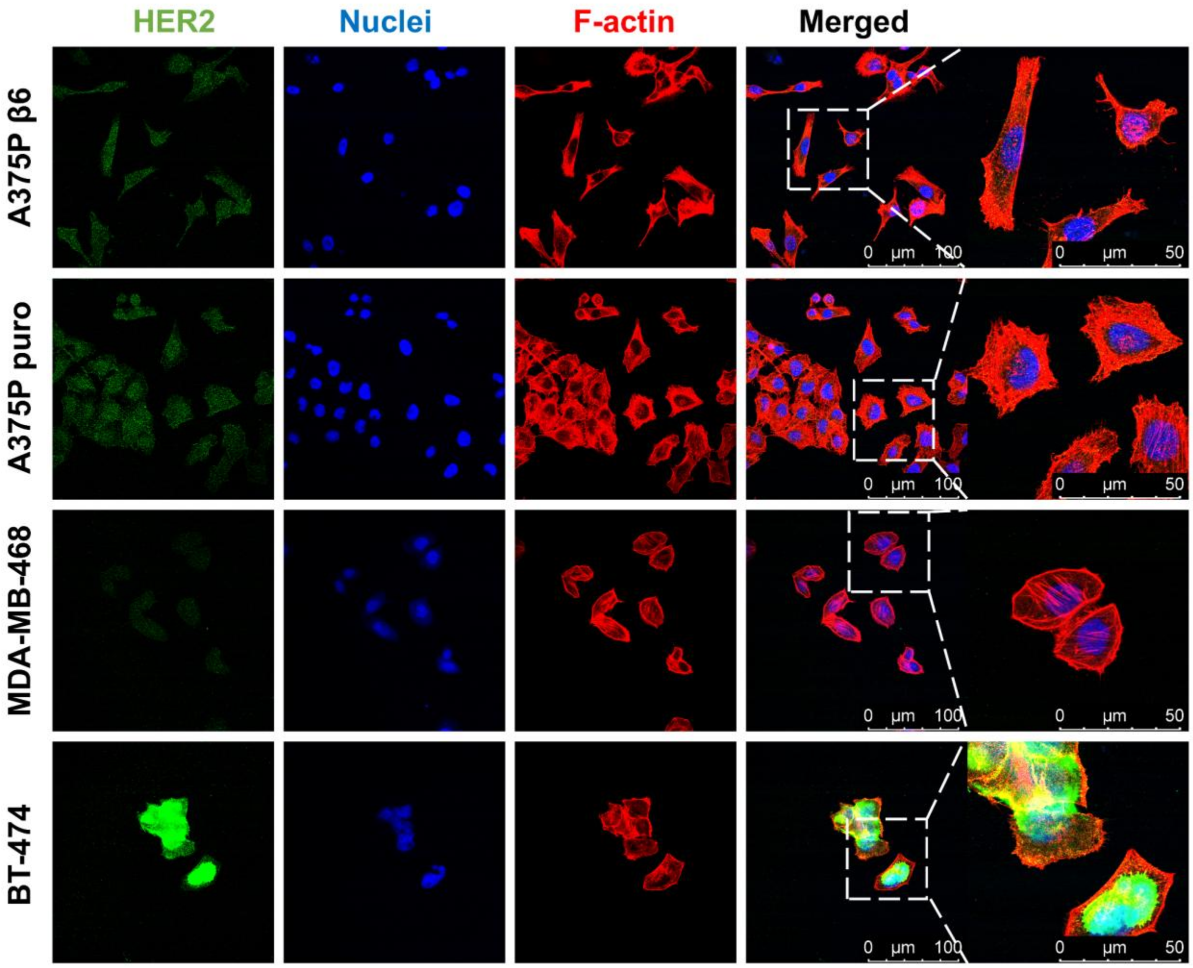

Figure S7. Immunofluorescence analysis of HER2 expression in A375P β6, A375P puro, MDA-MB-468, and BT-474 cells. Single z slice shown at the juxtamembrane region. Scale bar, 50 µm. Green (HER2); nuclei (blue); F-actin (red).

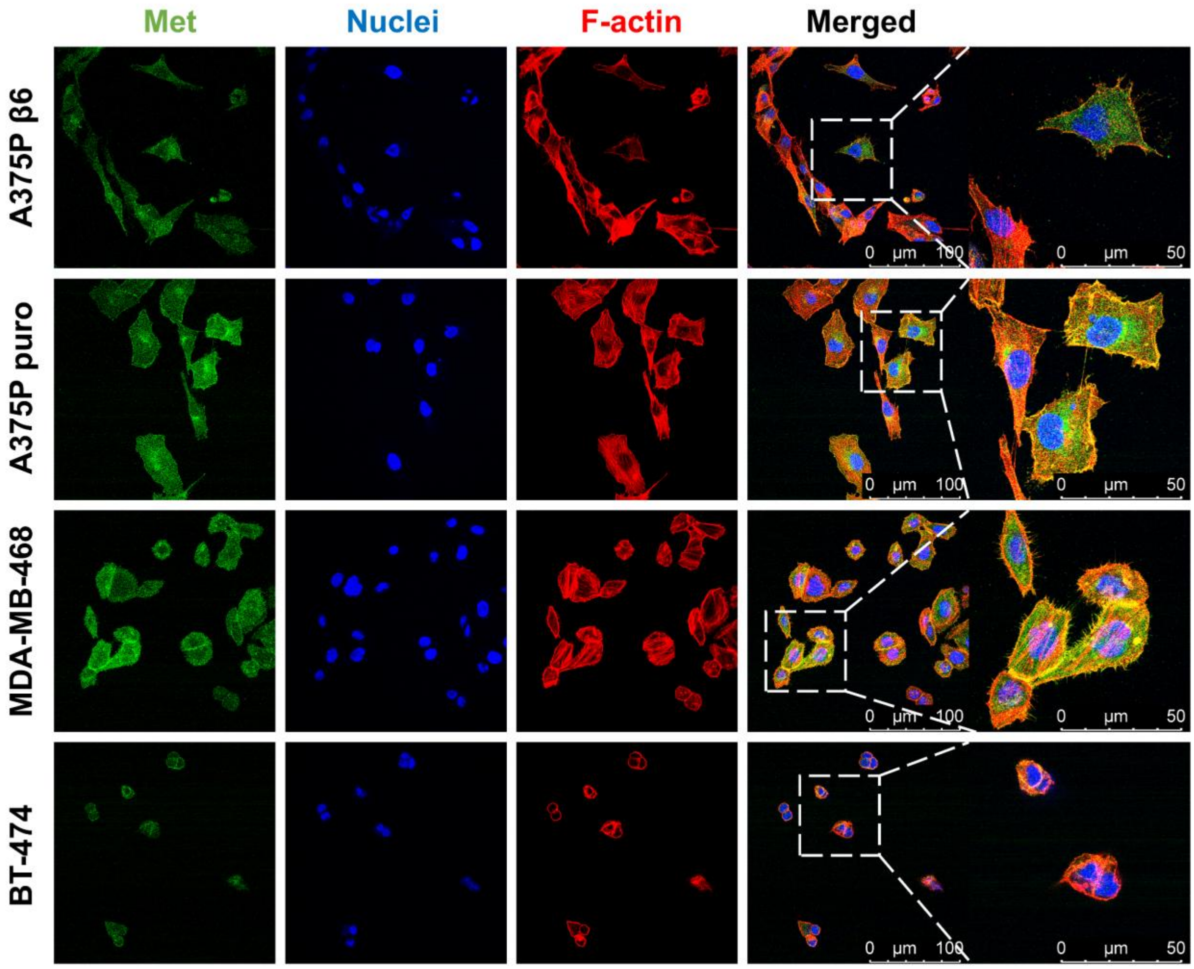

Figure S8. Immunofluorescence analysis of Met expression in A375P β6, A375P puro, MDA-MB-468, and BT-474 cells. Single z slice shown at the juxtamembrane region. Scale bar, 50 µm. Green (Met); nuclei (blue); F-actin (red).

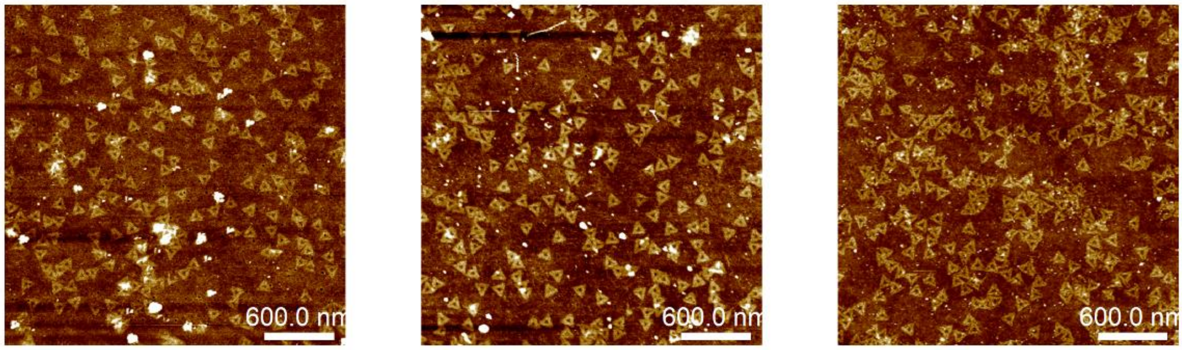

Figure S9. Density of DNA origami immobilized on the coverslip. The density is 29 ± 5 DNA origami/µm^2 (calculated from three repeated areas).

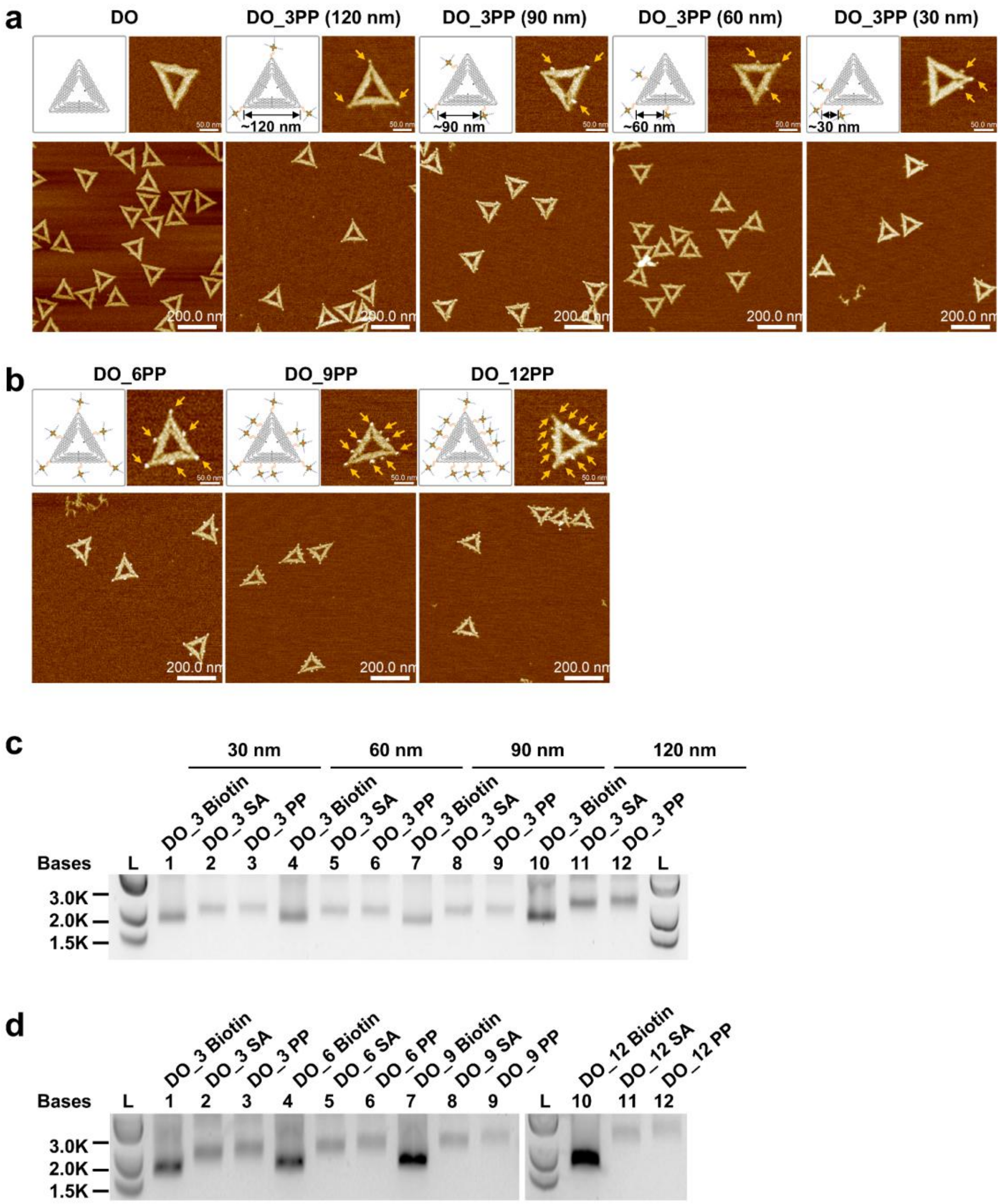

Figure S10. AFM and gel analysis of A20FMDV2 peptides functionalized DNA origami. Topographical AFM images of DNA origami functionalized with A20FMDV2 peptides at defined spacings (a) and densities (b). (c) Agarose gel analysis of DNA origami functionalized with A20FMDV2 peptides at four different spacings (30, 60, 90, and 120 nm). For each spacing, three conditions were analyzed: Lanes 1, 4, 7, 10: DNA origami with 3 biotin groups; Lanes 2, 5, 8, 11: DNA origami with 3 streptavidin (SA) molecules, showing slower migration than the corresponding biotin samples, indicating successful streptavidin binding; Lanes 3, 6, 9, 12: DNA origami with peptides, showing slightly slower migration than the corresponding streptavidin samples, confirming peptide attachment *via* streptavidin. (d) Agarose gel analysis

of DNA origami functionalized with A20FMDV2 peptides at four different ligand densities (3, 6, 9, and 12 peptides per origami). For each density, three conditions were analyzed: Lanes 1, 4, 7, 10: DNA origami with increasing numbers of biotin groups (3, 6, 9, or 12); Lanes 2, 5, 8, 11: Corresponding streptavidin-bound structures, showing slower migration than biotin-only samples; Lanes 3, 6, 9, 12: Peptide-conjugated structures, showing slightly slower migration than the streptavidin samples, confirming successful peptide attachment.

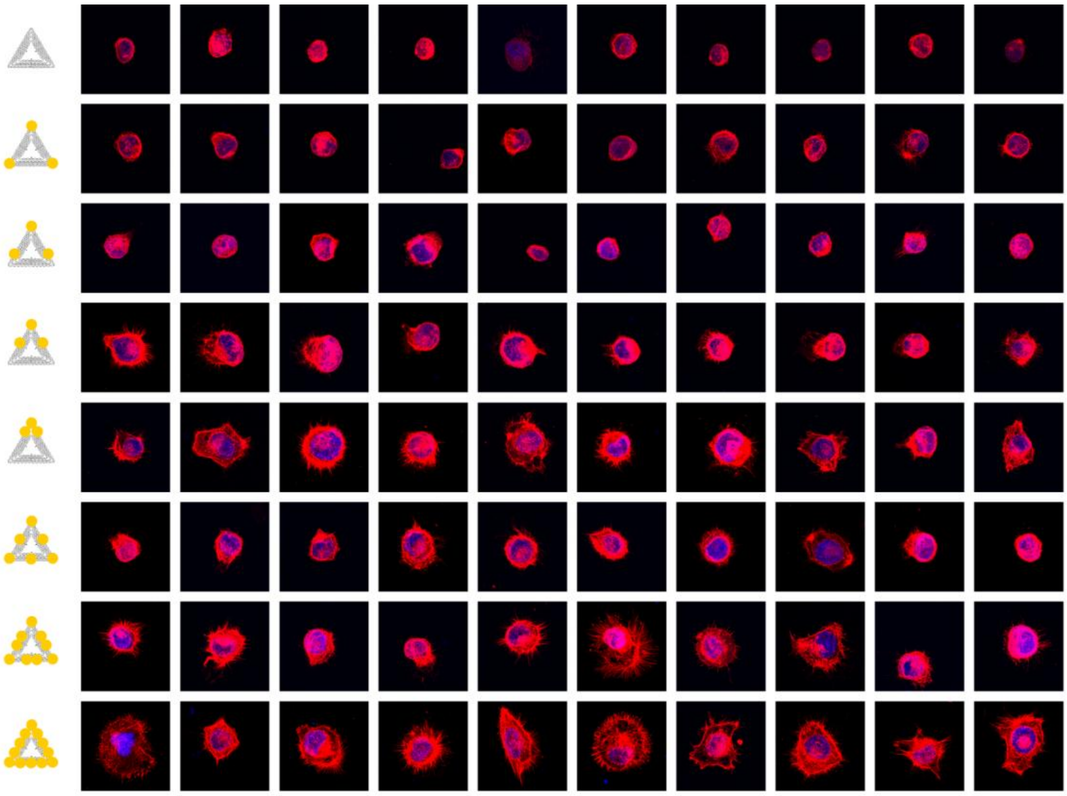

Figure S11. Z-projected confocal images of A375P β6 cells on peptides-functionalized substrates. F-actin (red); nuclei (blue). Regions of interest (ROIs, 46 μm × 46 μm) containing single cells were selected, and 40-layer confocal z-stacks were acquired from top to bottom of each cell.

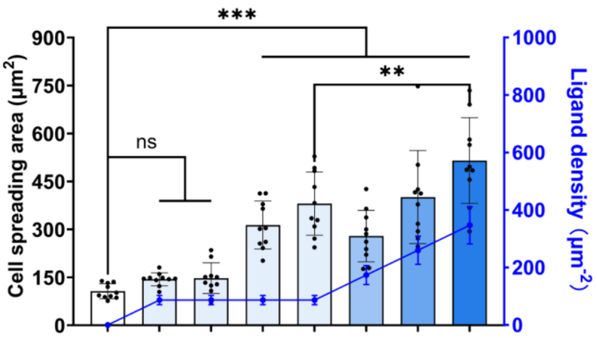

Number per DO	0	3	3	3	3	6	9	12
Spacing (nm)	-	120	90	60	30	60	40	30

Figure S12. Quantitative morphometric analysis of A375P β6 cells spreading on peptides-functionalized substrates. Left Y-axis: Quantification of projected cell area spreading on various peptides spacings- and densities- functionalized substrates (Morphometric data were obtained from images shown in **Figure S11**; one-way ANOVA, n = 10, **P <0.01, *** P <0.001; ns, not significant). Right Y-axis: corresponding A20FMDV2 peptide densities of substrates. Data represent mean ± SD.

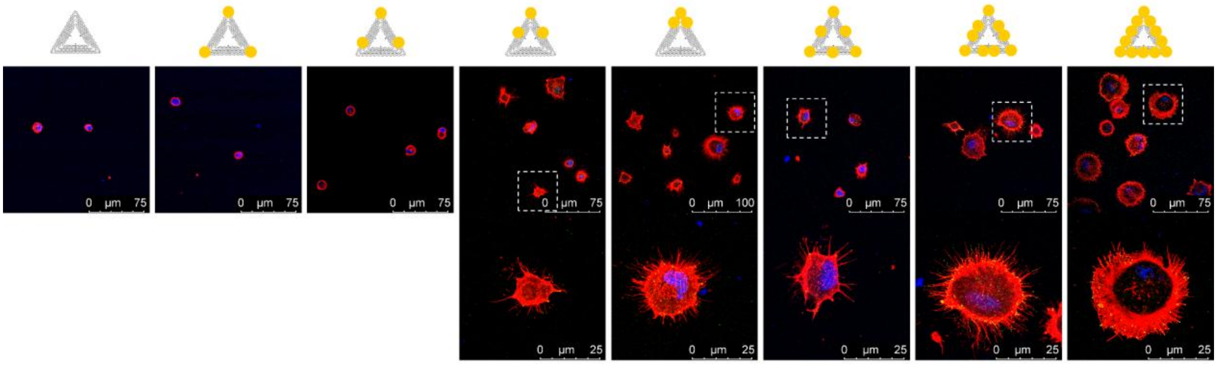

Figure S13. Larger field-of-view images of p-FAK in A375P β6 cells on peptides-functionalized substrates. Single z-slice at the juxtamembrane region. Scale bars: 75 μm (top), 25 μm (bottom). p-FAK (Y397, green); F-actin (red); nuclei (blue).

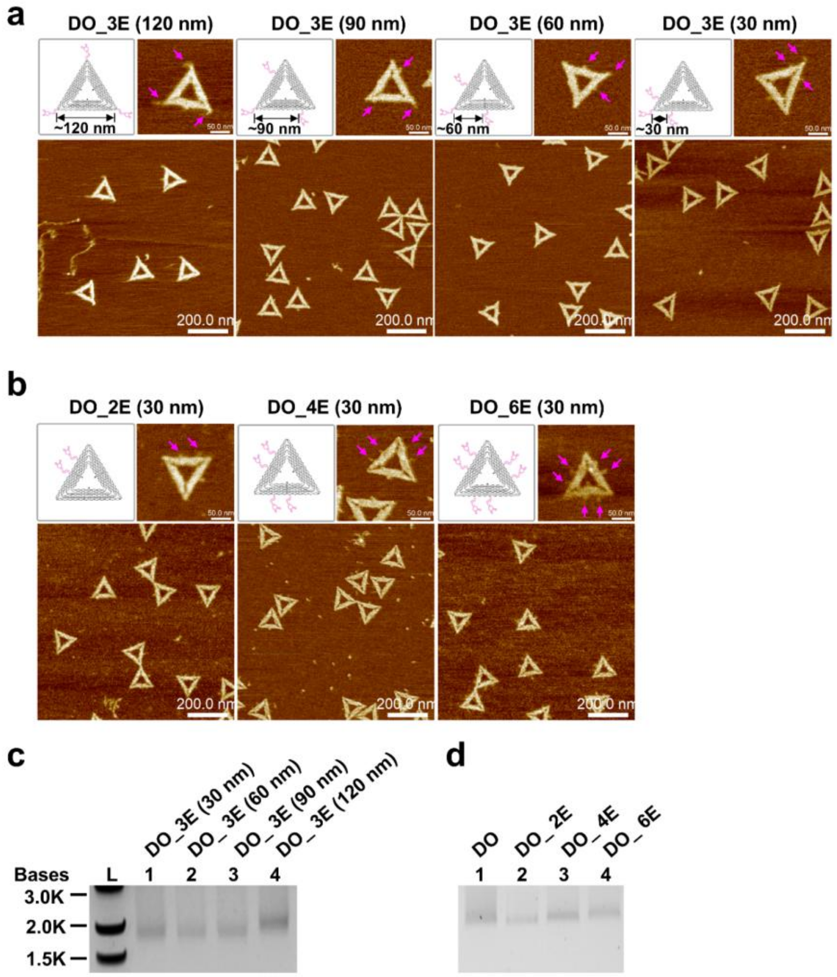

Figure S14. AFM and gel analysis of EGFR aptamers-functionalized DNA origami. Topographical AFM images of DNA origami functionalized with EGFR aptamers at defined spacings (a) and densities (b). (c) Agarose gel analysis of DNA origami functionalized with EGFR aptamers at different spacings. Lane 1: DNA origami with 3 EGFR aptamers spaced at 30 nm (DO_3E (30 nm)); Lane 2: 3 EGFR aptamers at 60 nm spacing (DO_3E (60 nm)); Lane 3: 3 EGFR aptamers at 90 nm spacing (DO_3E (90 nm)); Lane 4: 3 EGFR aptamers at 120 nm spacing (DO_3E (120 nm)). In the DO_3E (120 nm) design, the aptamers are positioned at the three vertices of the DNA triangle, resulting in a larger spatial configuration. Although the molecular weight is same across all samples, the more extended geometry of the 120 nm spacing leads to significantly slower gel migration compared to the other designs. (d) Agarose gel analysis of DNA origami functionalized with EGFR aptamers at different densities. Lane 1: DNA origami without aptamers; Lane 2: DNA origami with 2 EGFR aptamers (DO_2E); Lane 3: DNA origami with 4 EGFR aptamers (DO_4E); Lane 4: DNA origami with 6 EGFR aptamers (DO_6E). A progressive decrease in gel mobility was observed with increasing aptamer density, indicating successful modification and increased molecular mass.

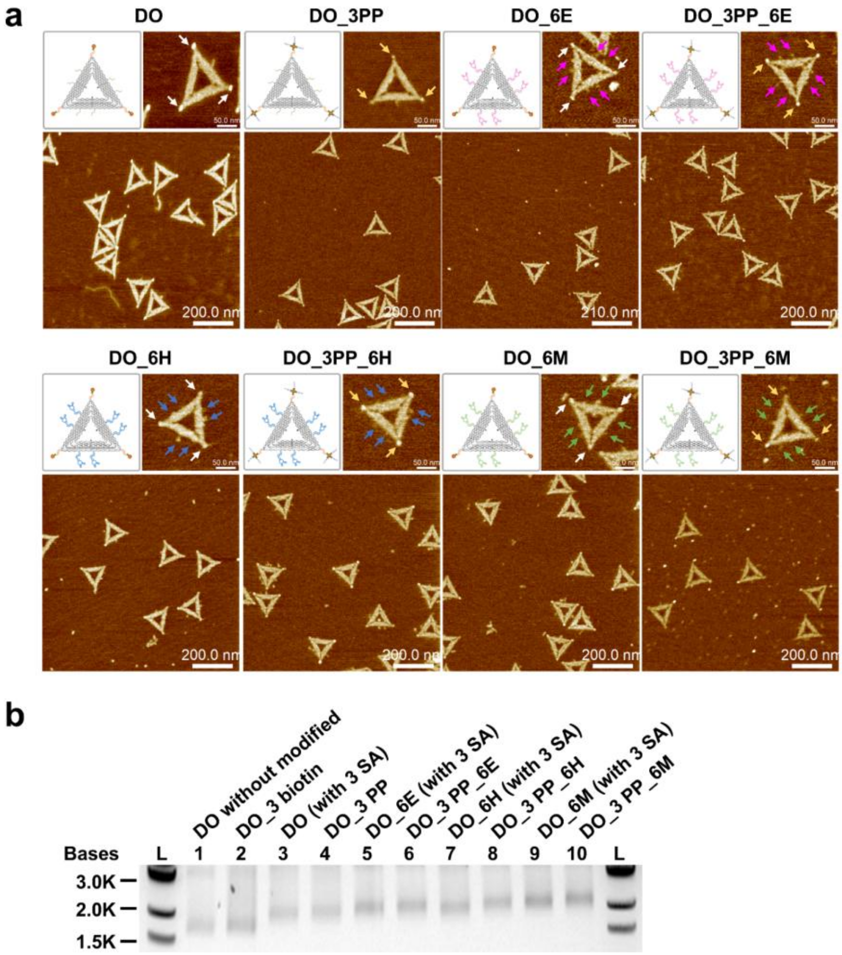

Figure S15. AFM and gel analysis of hetero-ligand functionalized DNA origami. (a) Topographical AFM images of peptide- and aptamer-functionalized DNA origami. (b) Agarose gel electrophoresis analysis of DNA origami functionalized with peptides and aptamers. Lane 1: DNA origami without modification; Lane 2: DNA origami with 3 biotins; Lane 3: DNA origami with 3 streptavidin (DO); Lane 4: DNA origami with 3 peptides (DO_3PP); Lane 5: DNA origami with 3 streptavidin and 6 EGFR aptamers (DO_6E); Lane 6: DNA origami with 3 peptides and 6 EGFR aptamers (DO_3PP_6E); Lane 7: DNA origami with 3 streptavidin and 6 HER2 aptamers (DO_6H); Lane 8: DNA origami with 3 peptides and 6 HER2 aptamers (DO_3PP_6H); Lane 9: DNA origami with 3 streptavidin and 6 Met aptamers (DO_6M); Lane 10: DNA origami with 3 peptides and 6 Met aptamers (DO_3PP_6M). Slower migration in modified samples confirms stepwise assembly of peptides and aptamers on the DNA origami scaffold.

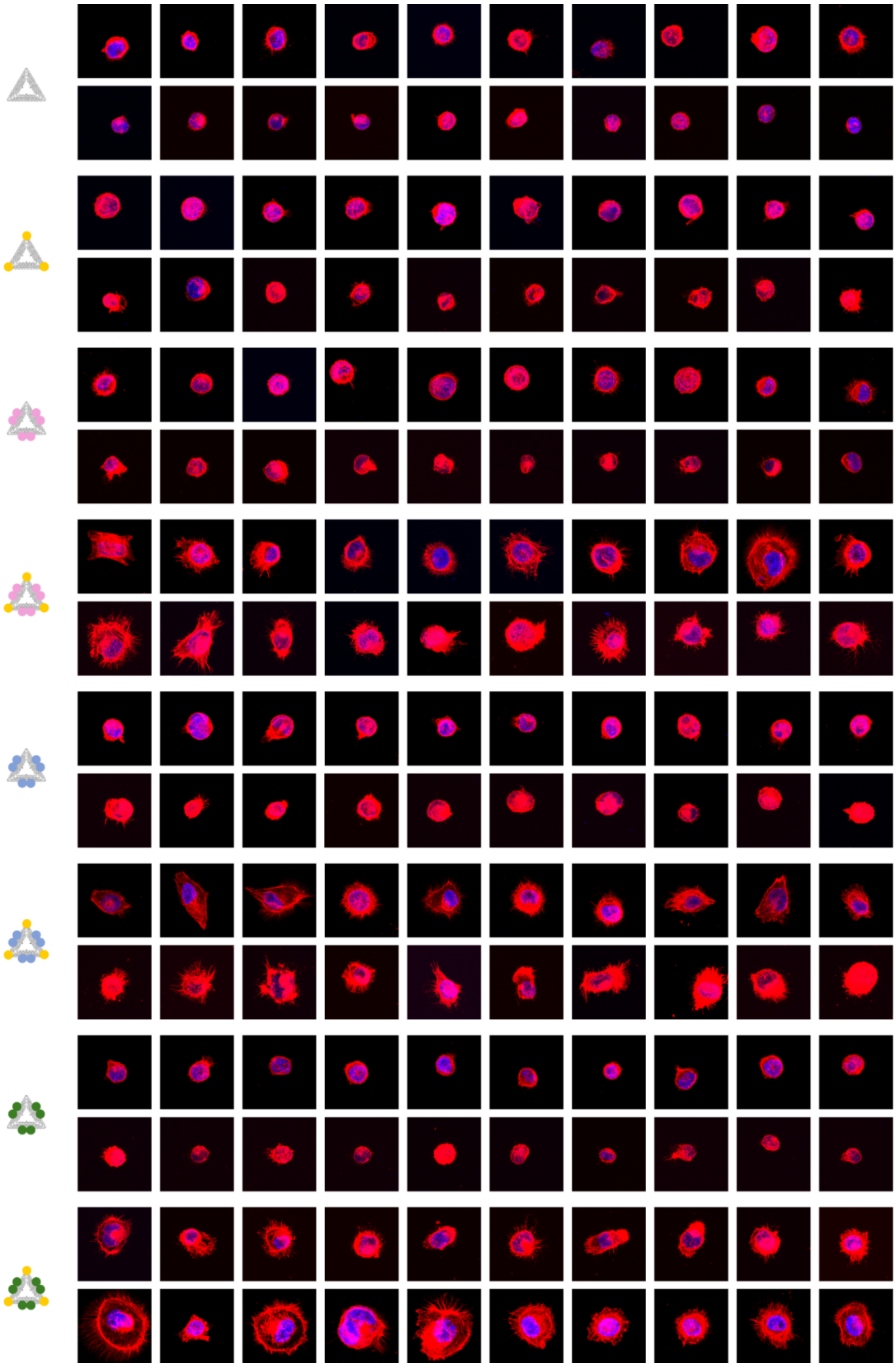

Figure S16. Z-projected confocal images of A375P β6 cells on hetero-ligand functionalized substrates. F-actin (red); nuclei (blue). ROIs (46 μm × 46 μm) containing single cells were selected, and 40-layer confocal z-stacks were acquired from top to bottom of each cell. Images were obtained from two independent experiments.

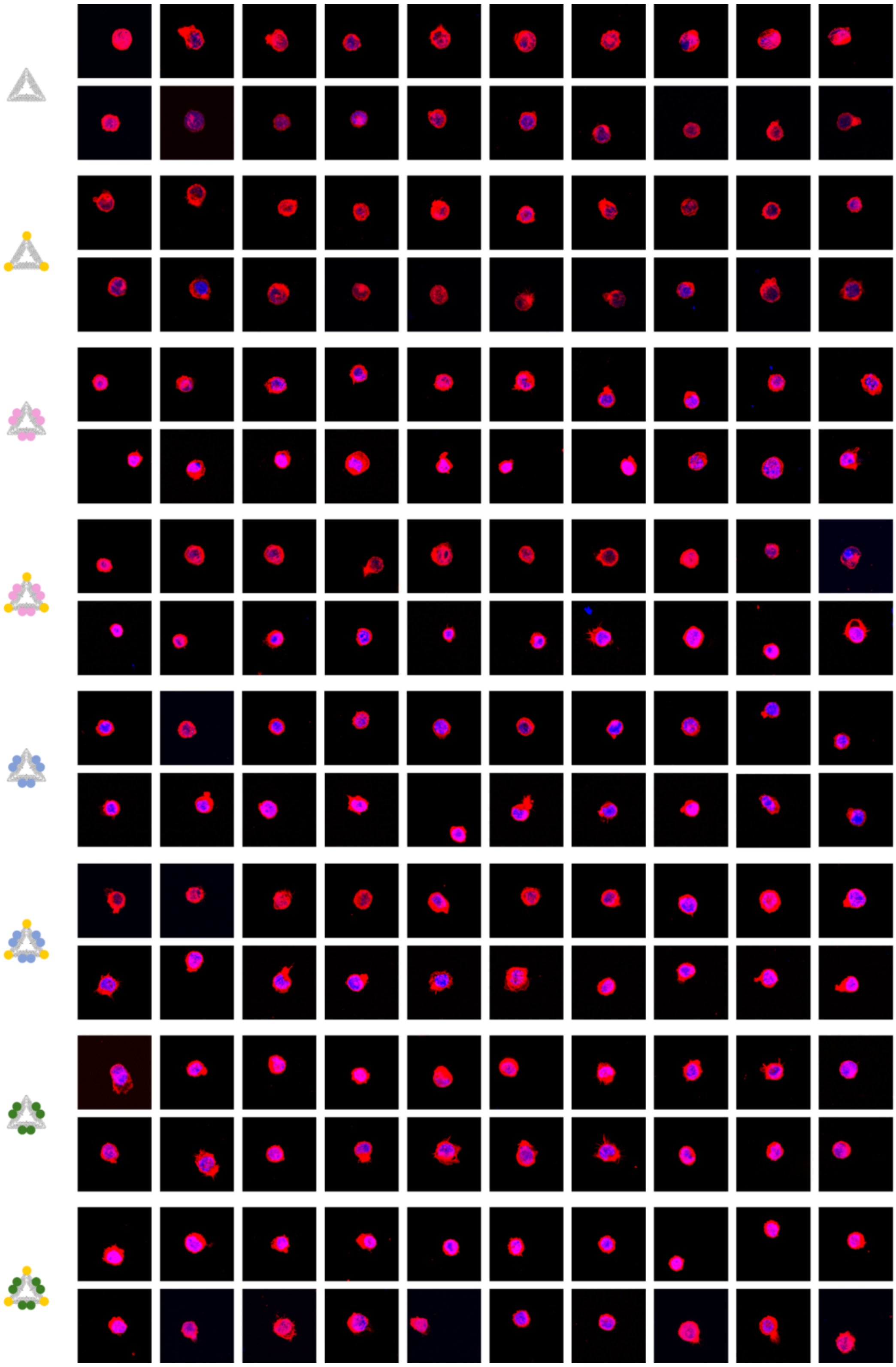

Figure S17. Z-projected confocal images of A375P puro cells on hetero-ligand functionalized substrates. F-actin (red); nuclei (blue). ROIs (46 µm × 46 µm) containing single cells were selected, and 40-layer confocal z-stacks were acquired from top to bottom of each cell. Images were obtained from two independent experiments.

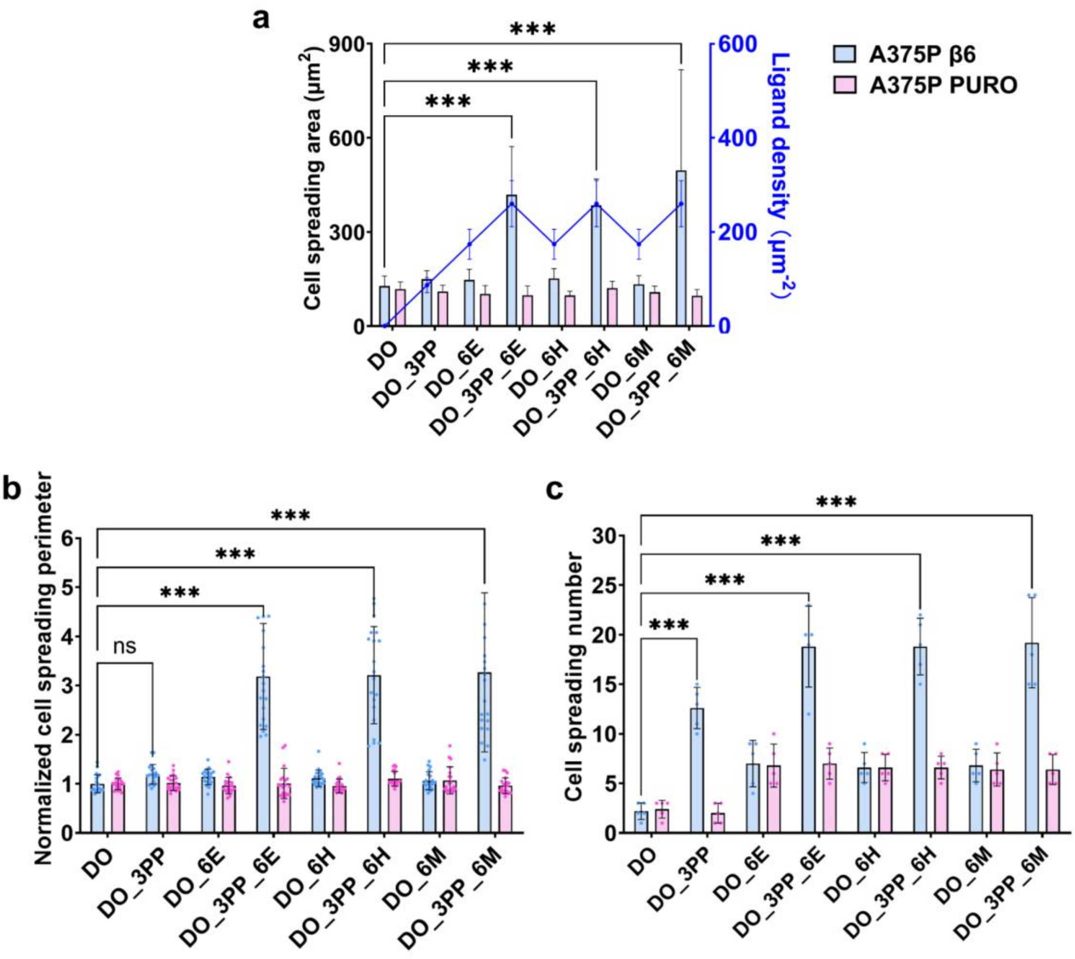

Figure S18. Single-cell quantitative morphometric analysis of A375P β6 and puro cells, showing the impact of ligand crosstalk on (a) cell spreading area, (b) perimeter and (c) spreading cell number, with DO as control (two-way ANOVA, ***P <0.001, ns: not significant). Data represent mean ± SD. Morphometric data (cell spreading area and perimeter) were obtained from images shown in **Figure S16** and **Figure S17** from two independent experiments. Spreading cell numbers were obtained from five different areas (300 μm × 300 μm).

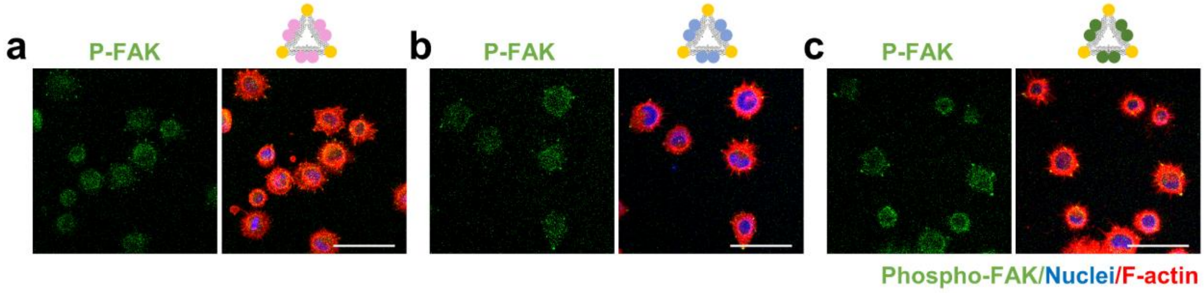

Figure S19. Larger field-of-view Z-projected confocal images of p-FAK in A375P β6 cells on hetero-ligand functionalized substrates. (a) DO_3PP_6E, (b) DO_3PP_6H, (c) DO_3PP_6M. p-FAK (green); F-actin (red); nuclei (blue). Scale bars: 40 μm.

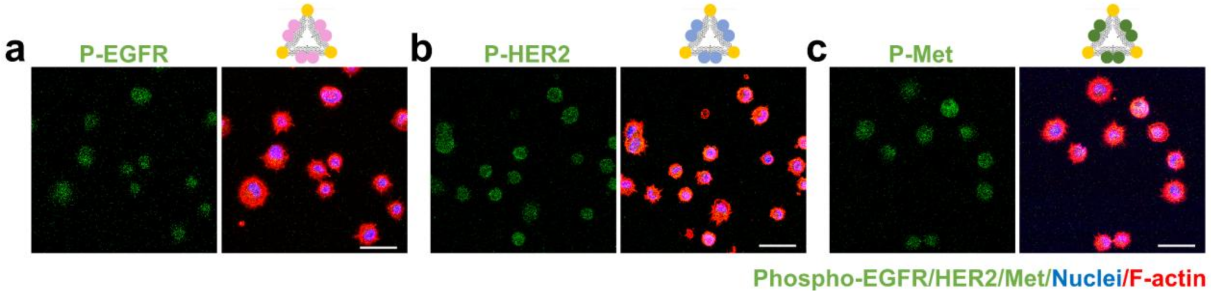

Figure S20. Larger field-of-view Z-projected confocal images of p-EGFR/p-HER2/p-Met in A375P β6 cells on hetero-ligand functionalized substrates. (a) DO_3PP_6E, (b) DO_3PP_6H, (c) DO_3PP_6M. p-EGFR/p-HER2/p-Met (green); F-actin (red); nuclei (blue). Scale bars: 40 μm.

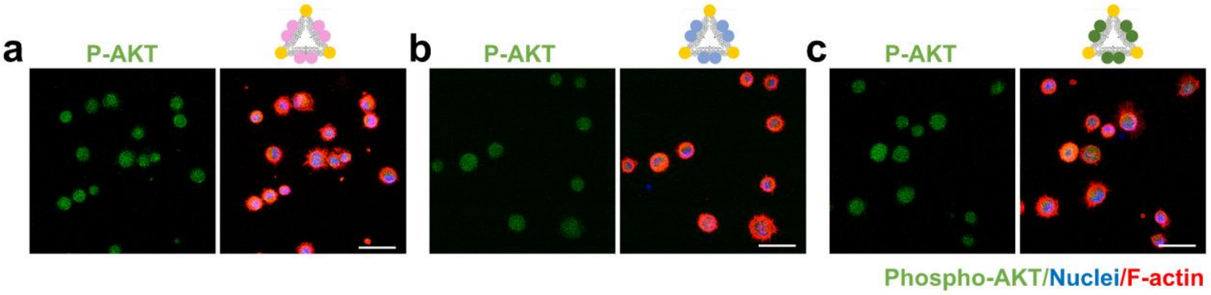

Figure S21. Larger field-of-view Z-projected confocal images of p-AKT in A375P β6 cells on hetero-ligand functionalized substrates. (a) DO_3PP_6E, (b) DO_3PP_6H, (c) DO_3PP_6M. p-AKT (green); F-actin (red); nuclei (blue). Scale bars: 40 μm.

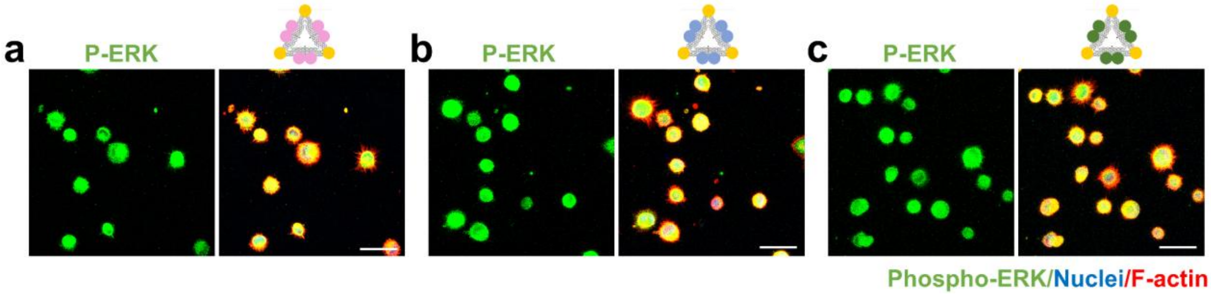

Figure S22. Larger field-of-view Z-projected confocal images of p-ERK in A375P β6 cells on hetero-ligand functionalized substrates. (a) DO_3PP_6E, (b) DO_3PP_6H, (c) DO_3PP_6M. p-ERK (green); F-actin (red); nuclei (blue). Scale bars: 40 μm.

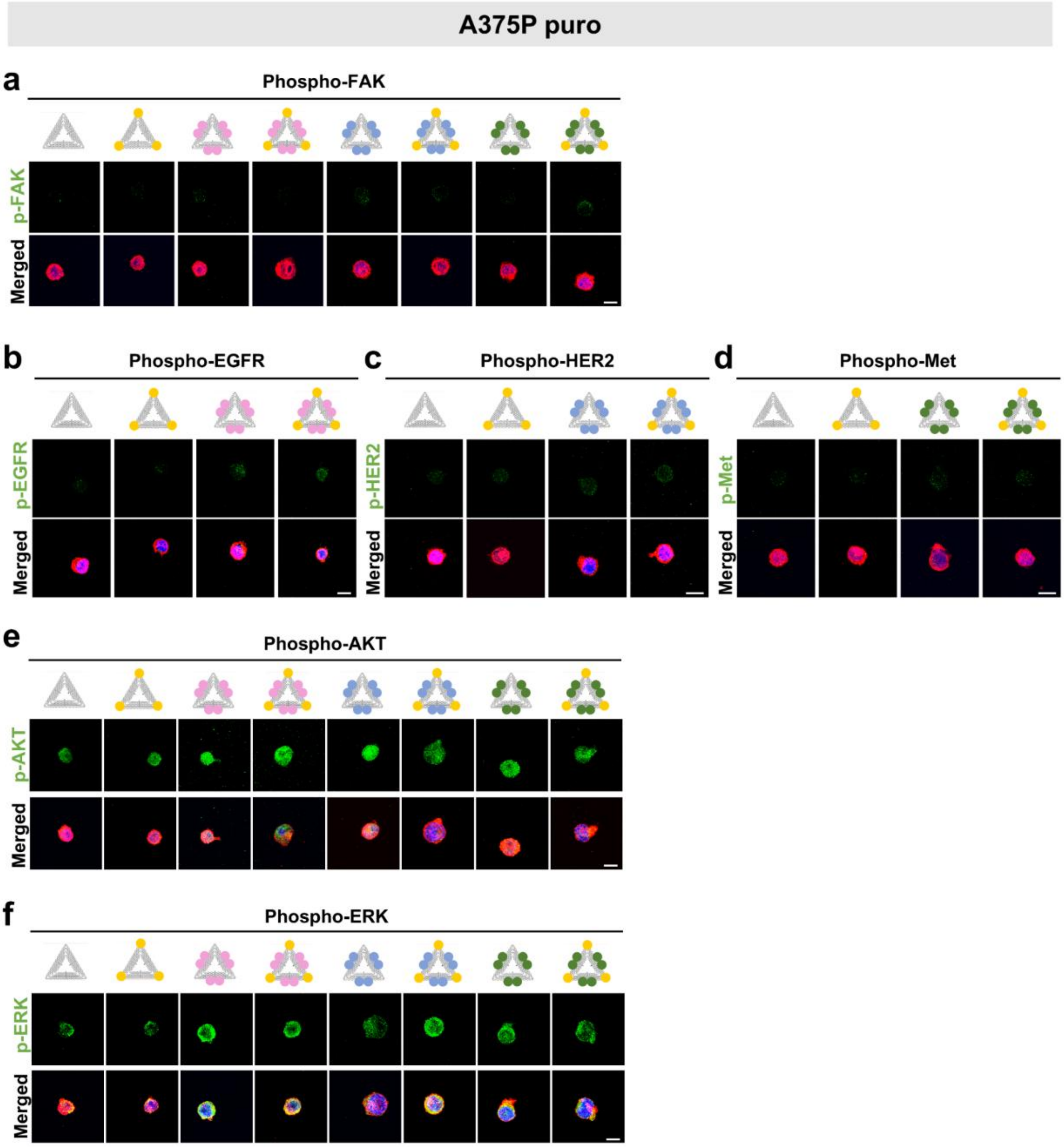

Figure S23. Phosphorylation signaling in A375P puro cells on functionalized substrates. Z-projected confocal images of A375P puro cells showing phosphorylation of FAK (a), EGFR (b), HER2 (c), Met (d), AKT (e) and ERK (f) (green) during cell spreading on various functionalized DNA origami substrates. F-actin (red); nuclei (blue). Scale bar, 10 µm.

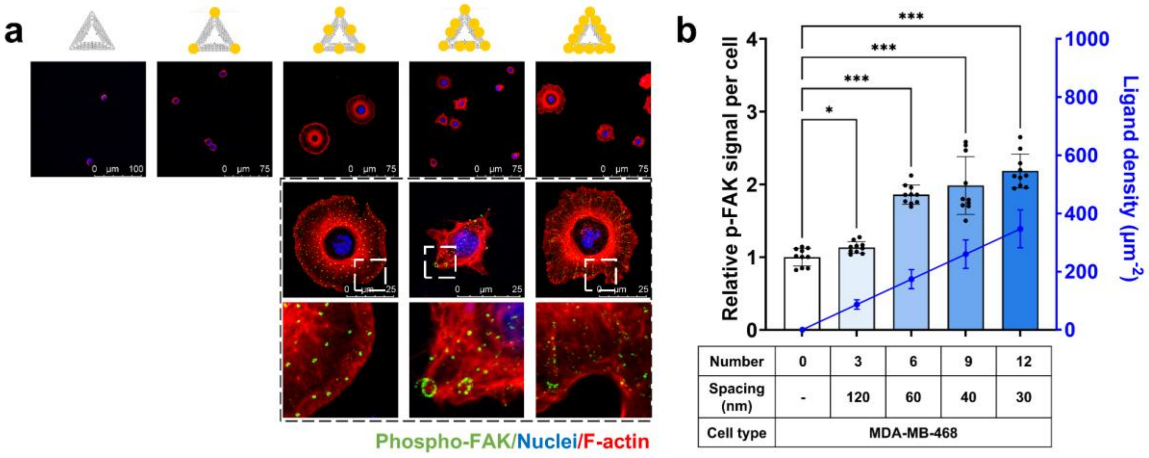

Figure S24. Quantitative morphometric analysis of MDA-MB-468 cells spreading on peptides functionalized substrates. (a) Confocal images of MDA-MB-468 cells spreading on substrates functionalized with varying densities of A20FMDV2 peptides. p-FAK (Y397, green); F-actin (red); nuclei (blue). (b) Left Y-axis: normalized p-FAK intensity in MDA-MB-468 cells, with DO as control (one-way ANOVA, n = 10, *P <0.05, ***P <0.001). Right Y-axis: corresponding A20FMDV2 peptides density of substrates. Data represent mean ± SD.

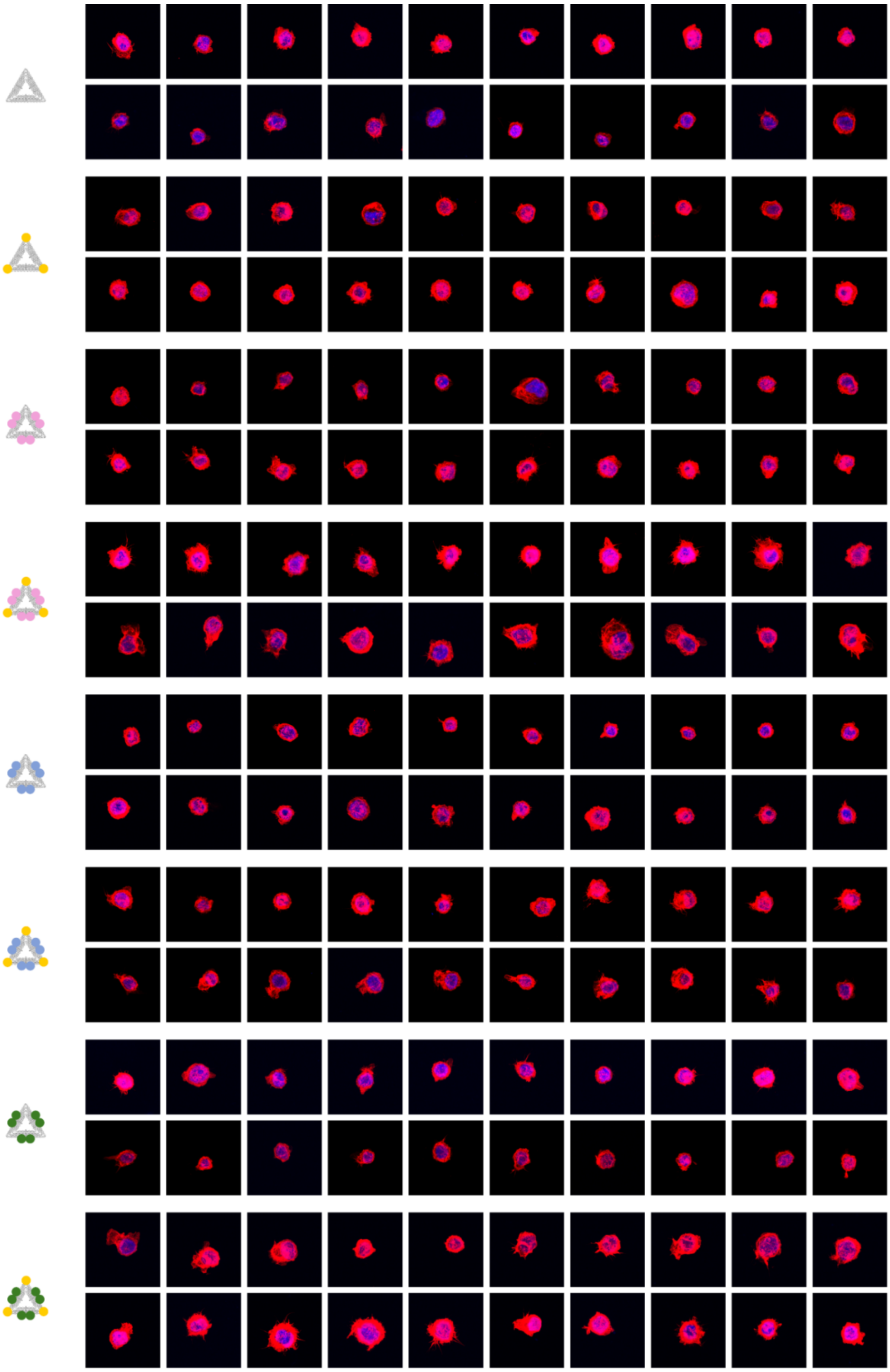

Figure S25. Z-projected confocal images of MDA-MB-468 cells on hetero-ligand functionalized substrates. F-actin (red); nuclei (blue). ROIs (46 μm × 46 μm) containing single cells were selected, and 40-layer confocal z-stacks were acquired from top to bottom of each cell. Images were obtained from two independent experiments.

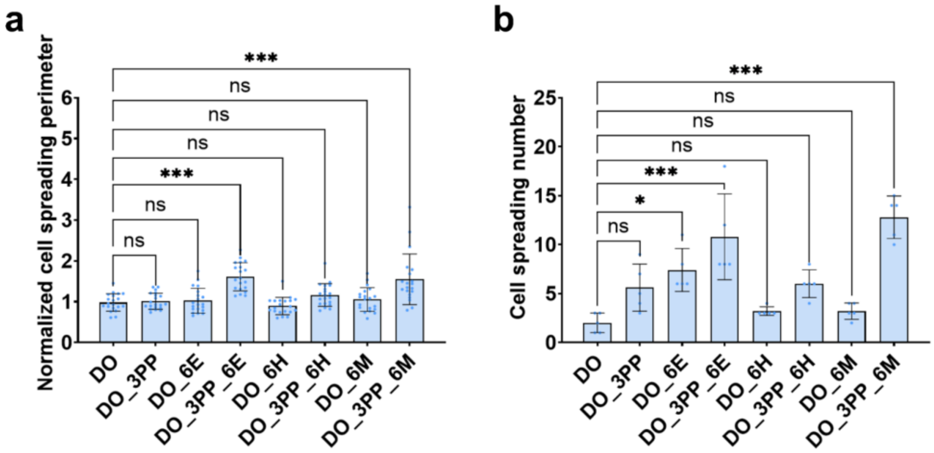

Figure S26. Quantitative morphometric analysis of MDA-MB-468 on hetero-ligand functionalized substrates, showing the impact of ligand crosstalk on cells spreading perimeter (a) and spreading cell number (b), with DO as control. (one-way ANOVA *P <0.05, ***P <0.001, ns: not significant). Data represent mean ± SD. Morphometric data (cell spreading perimeter) were obtained from images shown in **Figure S25** from two independent experiments. Spreading cell numbers were obtained from five different areas (300 µm × 300 µm).

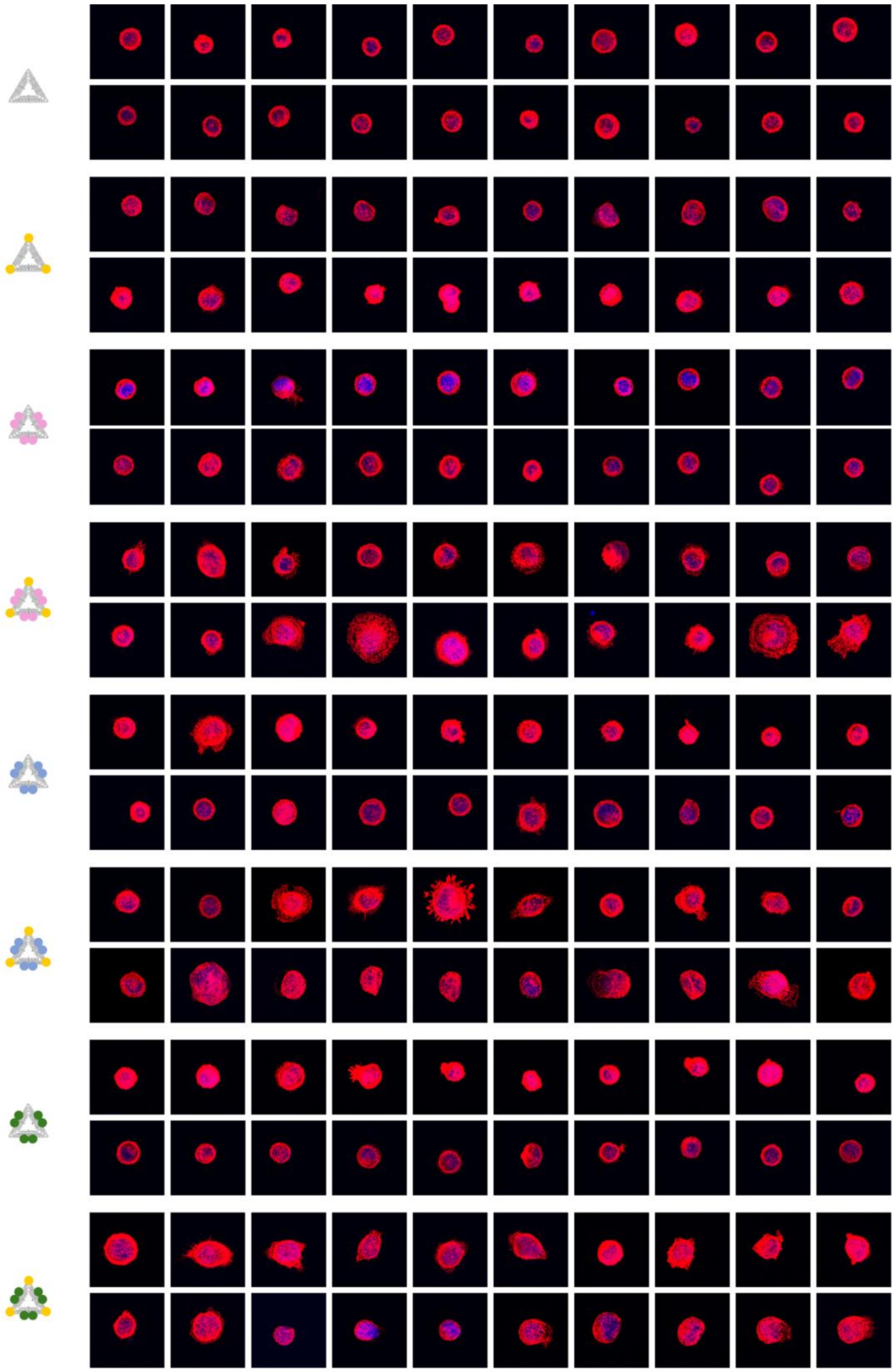

Figure S27. Z-projected confocal images of BT-474 cells on hetero-ligand functionalized substrates. F-actin (red); nuclei (blue). ROIs (46 μm × 46 μm) containing single cells were selected, and 40-layer confocal z-stacks were acquired from top to bottom of each cell. Images were obtained from two independent experiments.

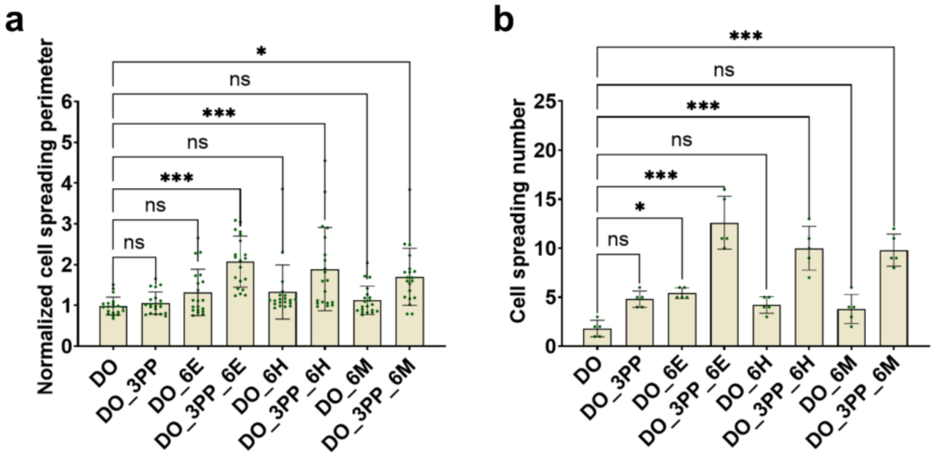

Figure S28. Quantitative morphometric analysis of BT-474 on hetero-ligand functionalized substrates, showing the impact of ligand crosstalk on cells spreading perimeter (a) and spreading cell number (b), with DO as control. (one-way ANOVA *P <0.05, ***P <0.001, ns: not significant). Data represent mean ± SD. Morphometric data (cell spreading perimeter) were obtained from images shown in **Figure S27** from two independent experiments. Spreading cell numbers were obtained from five different areas (300 μm × 300 μm).

Supporting Tables

Table S1. Sequences of amino anchors

A5-Amino	TTTGATGATTAAGAGGCTGAGACTTGCTCAGTACCAGGCGCCG ATTCAG-Amino
B5-Amino	ACAGTCAAAGAGAATCGATGAACGACCCCGGTTGATAATCCCG ATTCAG-Amino
C5-Amino	TGGCAATTTTTAACGTCAGATGAAAACAATAACGGATTCGCCG ATTCAG-Amino
A13-Amino	TTTAACGGTTCGGAACCTATTATTAGGGTTGATATAAGTACCGA TTCAG-Amino
B13-Amino	CGTTCTAGTCAGGTCATTGCCTGACAGGAAGATTGTATAACCG ATTCAG-Amino
C13-Amino	GATTATACACAGAAATAAAGAAATACCAAGTTACAAAATCCCG ATTCAG-Amino
A33-Amino	CGCGTCTGATAGGAACGCCATCAACTTTTACACCGATTCAG-Amino
B33-Amino	AGGGATAGCTCAGAGCCACCACCCCATGTCAACCGATTCAG-Amino
C33-Amino	GATTATACACAGAAATAAAGAAATACCAAGTTCCGATTCAG-Amino
A42-Amino	AGAGTCAAAAATCAATATATGTGATGAAACAAACATCAAGCCG ATTCAG-Amino
B42-Amino	AGACGTTACCATGTACCGTAACACCCCTCAGAACCGCCACCCG ATTCAG-Amino
C42-Amino	GTAACCGTCTTTCATCAACATTAAAATTTTTGTTAAATCACCGA TTCAG-Amino
A50-Amino	ACATAGCGCTGTAAATCGTCGCTATTCATTTCAATTACCTCCGA TTCAG-Amino
B50-Amino	AGCGTAACTACAAACTACAACGCCTATCACCGTACTCAGGCCG ATTCAG-Amino
C50-Amino	GGATAGGTACCCGTCGGATTCTCCTAAACGTTAATATTTTCCGA TTCAG-Amino

Table S2. Sequences for EGFR aptamer patterning

A26 extend_1	CACCGTCACCTTATTACGCAGTATTGAGTTAAGCCCAATAGTTATGTAACTCTGATA
B26 extend_1	CGGATGGCACGAGAATGACCATAATCGTTTACCAGACGACGTTATGTAACTCTGATA
C26 extend_1	CTATTAGTATATCCAGAACAATATCAGGAACGGTACGCCAGTTATGTAACTCTGATA
A32 extend_1	CAGAAGGAAACCGAGGTTTTTAAGAAAAGTAAGCAGATAGCCGGTTATGTAACTCTGATA
B32 extend_1	AATACTGCGGAATCGTAGGGGGTAATAGTAAAATGTTTAGACTGTTATGTAACTCTGATA
C32 extend_1	TCTTTGATTAGTAATAGTCTGTCCATCACGCAAATTAACCGTTGTTATGTAACTCTGATA
A37 extend_1	AGAGAATAACATAAAAACAGGGAAGCGCATTAGTTATGTAACTCTGATA
B37 extend_1	ACAGGTAGAAAGATTCATCAGTTGAGATTTAGGTTATGTAACTCTGATA
C37 extend_1	CGAGAAAGGAAGGGAAGCGTACTATGGTTGCTGTTATGTAACTCTGATA
A56 extend_1	ACAAGAAAGCAAGCAAATCAGATAACAGCCATATTATTTAGTTATGTAACTCTGATA
B56 extend_1	CCAAGCGCAGGCGCATAGGCTGGCAGAACTGGCTCATTATGTTATGTAACTCTGATA
C56 extend_1	TTAATGAAGTTTGATGGTGGTTCCGAGGTGCCGTAAAGCAGTTATGTAACTCTGATA
EGFR aptamer	TACCAGTGCGATGCTCAGTGCCGTTTCTTCTCTTTCGCTTTTTTTGCTTTTGAGCATGCTGACGCATTCGGTTGACTATCAGAGTTACATAAC

Table S3. Sequences for A20FMDV2 peptide patterning

A26 extend_1	CACCGTCACCTTATTACGCAGTATTGAGTTAAGCCCAATAGTTATGTAACTCTGATA
B26 extend_1	CGGATGGCACGAGAATGACCATAATCGTTTACCAGACGACGTTATGTAACTCTGATA
C26 extend_1	CTATTAGTATATCCAGAACAATATCAGGAACGGTACGCCAGTTATGTAACTCTGATA
A37 extend_1	AGAGAATAACATAAAAACAGGGAAGCGCATTAGTTATGTAACTCTGATA
B37 extend_1	ACAGGTAGAAAGATTCATCAGTTGAGATTTAGGTTATGTAACTCTGATA
C37 extend_1	CGAGAAAGGAAGGGAAGCGTACTATGGTTGCTGTTATGTAACTCTGATA
A56 extend_1	ACAAGAAAGCAAGCAAATCAGATAACAGCCATATTATTTAGTTATGTAACTCTGATA
B56 extend_1	CCAAGCGCAGGCGCATAGGCTGGCAGAACTGGCTCATTATGTTATGTAACTCTGATA
C56 extend_1	TTAATGAAGTTTGATGGTGGTTCCGAGGTGCCGTAAAGCAGTTATGTAACTCTGATA
A32 extend_2	CAGAAGGAAACCGAGGTTTTTAAGAAAAGTAAGCAGATAGCCGAGTTGTGGATCCTACT
B32 extend_2	AATACTGCGGAATCGTAGGGGGTAATAGTAAAATGTTTAGACTAGTTGTGGATCCTACT
C32 extend_2	TCTTTGATTAGTAATAGTCTGTCCATCACGCAAATTAACCGTTAGTTGTGGATCCTACT
Biotin DNA_1	Biotin-TATCAGAGTTACATAAC
Biotin DNA_2	Biotin-AGTAGGATCCACAACT

Table S4. Sequences of A20FMDV2 peptide[1]

A20FMDV2	NK (biotin)VPNLRGDLQVLAQKVARTC

Table S5. Sequences for A20FMDV2 peptide and RTK aptamer co-patterning

A37 extend_1	AGAGAATAACATAAAAACAGGGAAGCGCATTAGTTATGTAACTCTGATA
B37 extend_1	ACAGGTAGAAAGATTCATCAGTTGAGATTTAGGTTATGTAACTCTGATA
C37 extend_1	CGAGAAAGGAAGGGAAGCGTACTATGGTTGCTGTTATGTAACTCTGATA
A56 extend_1	ACAAGAAAGCAAGCAAATCAGATAACAGCCATATTATTTAGTTATGTAACTCTGATA
B56 extend_1	CCAAGCGCAGGCGCATAGGCTGGCAGAACTGGCTCATTATGTTATGTAACTCTGATA
C56 extend_1	TTAATGAAGTTTGATGGTGGTTCCGAGGTGCCGTAAAGCAGTTATGTAACTCTGATA
A32 extend_2	CAGAAGGAAACCGAGGTTTTTAAGAAAAGTAAGCAGATAGCCGAGTTGTGGATCCTACT
B32 extend_2	AATACTGCGGAATCGTAGGGGGTAATAGTAAAATGTTTAGACTAGTTGTGGATCCTACT
C32 extend_2	TCTTTGATTAGTAATAGTCTGTCCATCACGCAAATTAACCGTTAGTTGTGGATCCTACT
Biotin DNA_2	Biotin-AGTAGGATCCACAACT
EGFR aptamer[2]	TACCAGTGCGATGCTCAGTGCCGTTTCTTCTCTTTCGCTTTTTTTGCTTTTGAGCATGCTGACGCATTCGGTTGACTATCAGAGTTACATAAC
HER2 aptamer[3]	GCAGCGGTGTGGGGGCAGCGGTGTGGGGGCAGCGGTGTGGGGTATCAGAGTTACATAAC
Met aptamer[4]	ATCAGGCTGGATGGTAGCTCGGTCGGGGTGGGTGGGTTGGCAAGTCTGATTATCAGAGTTACATAAC

Table S6. List of unmodified staple strands[5]

A01	CGGGGTTTCCTCAAGAGAAGGATTTTGAATTA
A02	AGCGTCATGTCTCTGAATTTACCGACTACCTT
A03	TTCATAATCCCCTTATTAGCGTTTTTCTTACC
A04	ATGGTTTATGTCACAATCAATAGATATTAAAC
A05	TTTGATGATTAAGAGGCTGAGACTTGCTCAGTACCAGGCG
A06	CCGGAACCCAGAATGGAAAGCGCAACATGGCT
A07	AAAGACAACATTTTCGGTCATAGCCAAAATCA
A08	GACGGGAGAATTAACTCGGAATAAGTTTATTTCCAGCGCC
A09	GATAAGTGCCGTCGAGCTGAAACATGAAAGTATACAGGAG
A10	TGTACTGGAAATCCTCATTAAAGCAGAGCCAC
A11	CACCGGAAAGCGCGTTTTCATCGGAAGGGCGA
A12	CATTCAACAAACGCAAAGACACCAGAACACCCTGAACAAA
A13	TTTAACGGTTCGGAACCTATTATTAGGGTTGATATAAGTA
A14	CTCAGAGCATATTCACAAACAAATTAATAAGT
A15	GGAGGGAATTTAGCGTCAGACTGTCCGCCTCC
A16	GTCAGAGGGTAATTGATGGCAACATATAAAAGCGATTGAG
A17	TAGCCCGGAATAGGTGAATGCCCCCTGCCTATGGTCAGTG
A18	CCTTGAGTCAGACGATTGGCCTTGCGCCACCC
A19	TCAGAACCCAGAATCAAGTTTGCCGGTAAATA
A20	TTGACGGAAATACATACATAAAGGGCGCTAATATCAGAGA
A21	CAGAGCCAGGAGGTTGAGGCAGGTAACAGTGCCCG
A22	ATTAAAGGCCGTAATCAGTAGCGAGCCACCCT
A23	GATAACCCACAAGAATGTTAGCAAACGTAGAAAATTATTC
A24	GCCGCCAGCATTGACACCACCCTC
A25	AGAGCCGCACCATCGATAGCAGCATGAATTAT
A26	CACCGTCACCTTATTACGCAGTATTGAGTTAAGCCCAATA
A27	AGCCATTTAAACGTCACCAATGAACACCAGAACCA
A28	ATAAGAGCAAGAAACATGGCATGATTAAGACTCCGACTTG
A29	CCATTAGCAAGGCCGGGGGAATTA
A30	GAGCCAGCGAATACCCAAAAGAACATGAAATAGCAATAGC
A31	TATCTTACCGAAGCCCAAACGCAATAATAACGAAAATCACCAG

A32	CAGAAGGAAACCGAGGTTTTAAGAAAAGTAAGCAGATAGCCG
A33	CCTTTTTTCATTTAACAATTTCATAGGATTAG
A34	TTTAACCTATCATAGGTCTGAGAGTTCCAGTA
A35	AGTATAAAATATGCGTTATACAAAGCCATCTT
A36	CAAGTACCTCATTCCAAGAACGGGAAATTCAT
A37	AGAGAATAACATAAAAACAGGGAAGCGCATTA
A38	AAAACAAAATTAATTAAATGGAAACAGTACATTAGTGAAT
A39	TTATCAAACCGGCTTAGGTTGGGTAAGCCTGT
A40	TTAGTATCGCCAACGCTCAACAGTCGGCTGTC
A41	TTTCCTTAGCACTCATCGAGAACAATAGCAGCCTTTACAG
A42	AGAGTCAAAAATCAATATATGTGATGAAACAAACATCAAG
A43	ACTAGAAATATATAACTATATGTACGCTGAGA
A44	TCAATAATAGGGCTTAATTGAGAATCATAATT
A45	AACGTCAAAAATGAAAAGCAAGCCGTTTTTATGAAACCAA
A46	GAGCAAAAGAAGATGAGTGAATAACCTTGCTTATAGCTTA
A47	GATTAAGAAATGCTGATGCAAATCAGAATAAA
A48	CACCGGAATCGCCATATTTAACAAAATTTACG
A49	AGCATGTATTTCATCGTAGGAATCAAACGATTTTTTGTTT
A50	ACATAGCGCTGTAAATCGTCGCTATTCATTTCAATTACCT
A51	GTTAAATACAATCGCAAGACAAAGCCTTGAAA
A52	CCCATCCTCGCCAACATGTAATTTAATAAGGC
A53	TCCCAATCCAAATAAGATTACCGCGCCCAATAAATAATAT
A54	TCCCTTAGAATAACGCGAGAAAACTTTTACCGACC
A55	GTGTGATAAGGCAGAGGCATTTTCAGTCCTGA
A56	ACAAGAAAGCAAGCAAATCAGATAACAGCCATATTATTTA
A57	GTTTGAAATTCAAATATATTTTAG
A58	AATAGATAGAGCCAGTAATAAGAGATTTAATG
A59	GCCAGTTACAAAATAATAGAAGGCTTATCCGGTTATCAAC
A60	TTCTGACCTAAAATATAAAGTACCGACTGCAGAAC
A61	GCGCCTGTTATTCTAAGAACGCGATTCCAGAGCCTAATTT
A62	TCAGCTAAAAAAGGTAAAGTAATT
A63	ACGCTAACGAGCGTCTGGCGTTTTAGCGAACCCAACATGT

A64	ACGACAATAAATCCCGACTTGCGGGAGATCCTGAATCTTACCA
A65	TGCTATTTTGCACCCAGCTACAATTTTGTTTTGAAGCCTTAAA
B01	TCATATGTGTAATCGTAAAACTAGTCATTTTC
B02	GTGAGAAAATGTGTAGGTAAAGATACAACTTT
B03	GGCATCAAATTTGGGGCGCGAGCTAGTTAAAG
B04	TTCGAGCTAAGACTTCAAATATCGGGAACGAG
B05	ACAGTCAAAGAGAATCGATGAACGACCCCGGTTGATAATC
B06	ATAGTAGTATGCAATGCCTGAGTAGGCCGGAG
B07	AACCAGACGTTTAGCTATATTTTCTTCTACTA
B08	GAATACCACATTCAACTTAAGAGGAAGCCCGATCAAAGCG
B09	AGAAAAGCCCCAAAAAGAGTCTGGAGCAAACAATCACCAT
B10	CAATATGACCCTCATATATTTTAAAGCATTAA
B11	CATCCAATAAATGGTCAATAACCTCGGAAGCA
B12	AACTCCAAGATTGCATCAAAAAGATAATGCAGATACATAA
B13	CGTTCTAGTCAGGTCATTGCCTGACAGGAAGATTGTATAA
B14	CAGGCAAGATAAAAATTTTTAGAATATTCAAC
B15	GATTAGAGATTAGATACATTTCGCAAATCATA
B16	CGCCAAAAGGAATTACAGTCAGAAGCAAAGCGCAGGTCAG
B17	GCAAATATTTAAATTGAGATCTACAAAGGCTACTGATAAA
B18	TTAATGCCTTATTTCAACGCAAGGGCAAAGAA
B19	TTAGCAAATAGATTTAGTTTGACCAGTACCTT
B20	TAATTGCTTTACCCTGACTATTATGAGGCATAGTAAGAGC
B21	ATAAAGCCTTTGCGGGAGAAGCCTGGAGAGGGTAG
B22	TAAGAGGTCAATTCTGCGAACGAGATTAAGCA
B23	AACACTATCATAACCCATCAAAAATCAGGTCTCCTTTTGA
B24	ATGACCCTGTAATACTTCAGAGCA
B25	TAAAGCTATATAACAGTTGATTCCCATTTTTG
B26	CGGATGGCACGAGAATGACCATAATCGTTTACCAGACGAC
B27	TAATTGCTTGGAAGTTTCATTCCAAATCGGTTGTA
B28	GATAAAAACCAAAATATTAAACAGTTCAGAAATTAGAGCT
B29	ACTAAAGTACGGTGTCGAATATAA
B30	TGCTGTAGATCCCCCTCAAATGCTGCGAGAGGCTTTTGCA

B31	AAAGAAGTTTTGCCAGCATAAATATTCATTGACTCAACATGTT
B32	AATACTGCGGAATCGTAGGGGGTAATAGTAAAATGTTTAGACT
B33	AGGGATAGCTCAGAGCCACCACCCCATGTCAA
B34	CAACAGTTTATGGGATTTTGCTAATCAAAAGG
B35	GCCGCTTTGCTGAGGCTTGCAGGGGAAAAGGT
B36	GCGCAGACTCCATGTTACTTAGCCCGTTTTAA
B37	ACAGGTAGAAAGATTCATCAGTTGAGATTTAG
B38	CCTCAGAACCGCCACCCAAGCCCAATAGGAACGTAAATGA
B39	ATTTTCTGTCAGCGGAGTGAGAATACCGATAT
B40	ATTCGGTCTGCGGGATCGTCACCCGAAATCCG
B41	CGACCTGCGGTCAATCATAAGGGAACGGAACAACATTATT
B42	AGACGTTACCATGTACCGTAACACCCCTCAGAACCGCCAC
B43	CACGCATAAGAAAGGAACAACTAAGTCTTTCC
B44	ATTGTGTCTCAGCAGCGAAAGACACCATCGCC
B45	TTAATAAAACGAACTAACCGAACTGACCAACTCCTGATAA
B46	AGGTTTAGTACCGCCATGAGTTTCGTCACCAGGATCTAAA
B47	GTTTTGTCAGGAATTGCGAATAATCCGACAAT
B48	GACAACAAGCATCGGAACGAGGGTGAGATTTG
B49	TATCATCGTTGAAAGAGGACAGATGGAAGAAAAATCTACG
B50	AGCGTAACTACAAACTACAACGCCTATCACCGTACTCAGG
B51	TAGTTGCGAATTTTTTCACGTTGATCATAGTT
B52	GTACAACGAGCAACGGCTACAGAGGATACCGA
B53	ACCAGTCAGGACGTTGGAACGGTGTACAGACCGAAACAAA
B54	ACAGACAGCCCAAATCTCCAAAAAAAAATTTCTTA
B55	AACAGCTTGCTTTGAGGACTAAAGCGATTATA
B56	CCAAGCGCAGGCGCATAGGCTGGCAGAACTGGCTCATTAT
B57	CGAGGTGAGGCTCCAAAAGGAGCC
B58	ACCCCCAGACTTTTTCATGAGGAACTTGCTTT
B59	ACCTTATGCGATTTTATGACCTTCATCAAGAGCATCTTTG
B60	CGGTTTATCAGGTTTCCATTAAACGGGAATACACT
B61	AAAACACTTAATCTTGACAAGAACTTAATCATTGTGAATT
B62	GGCAAAAGTAAAATACGTAATGCC

B63	TGGTTTAATTTCAACTCGGATATTCATTACCCACGAAAGA
B64	ACCAACCTAAAAAATCAACGTAACAAATAAATTGGGCTTGAGA
B65	CCTGACGAGAAACACCAGAACGAGTAGGCTGCTCATTCAGTGA
C01	TCGGGAGATATACAGTAACAGTACAAATAATT
C02	CCTGATTAAAGGAGCGGAATTATCTCGGCCTC
C03	GCAAATCACCTCAATCAATATCTGCAGGTCGA
C04	CGACCAGTACATTGGCAGATTCACCTGATTGC
C05	TGGCAATTTTTAACGTCAGATGAAAACAATAACGGATTCG
C06	AAGGAATTACAAAGAAACCACCAGTCAGATGA
C07	GGACATTCACCTCAAATATCAAACACAGTTGA
C08	TTGACGAGCACGTATACTGAAATGGATTATTTAATAAAAG
C09	CCTGATTGCTTTGAATTGCGTAGATTTTCAGGCATCAATA
C10	TAATCCTGATTATCATTTTGCGGAGAGGAAGG
C11	TTATCTAAAGCATCACCTTGCTGATGGCCAAC
C12	AGAGATAGTTTGACGCTCAATCGTACGTGCTTTCCTCGTT
C13	GATTATACACAGAAATAAAGAAATACCAAGTTACAAAATC
C14	TAGGAGCATAAAAGTTTGAGTAACATTGTTTG
C15	TGACCTGACAAATGAAAAATCTAAAATATCTT
C16	AGAATCAGAGCGGGAGATGGAAATACCTACATAACCCTTC
C17	GCGCAGAGGCGAATTAATTATTTGCACGTAAATTCTGAAT
C18	AATGGAAGCGAACGTTATTAATTTCTAACAAC
C19	TAATAGATCGCTGAGAGCCAGCAGAAGCGTAA
C20	GAATACGTAACAGGAAAAACGCTCCTAAACAGGAGGCCGA
C21	TCAATAGATATTAAATCCTTTGCCGGTTAGAACCT
C22	CAATATTTGCCTGCAACAGTGCCATAGAGCCG
C23	TTAAAGGGATTTTAGATACCGCCAGCCATTGCGGCACAGA
C24	ACAATTCGACAACTCGTAATACAT
C25	TTGAGGATGGTCAGTATTAACACCTTGAATGG
C26	CTATTAGTATATCCAGAACAATATCAGGAACGGTACGCCA
C27	CGCGAACTAAAACAGAGGTGAGGCTTAGAAGTATT
C28	GAATCCTGAGAAGTGTATCGGCCTTGCTGGTACTTTAATG
C29	ACCACCAGCAGAAGATGATAGCCC

C30	TAAAACATTAGAAGAACTCAAACTTTTTATAATCAGTGAG
C31	GCCACCGAGTAAAAGAACATCACTTGCCTGAGCGCCATTAAAA
C32	TCTTTGATTAGTAATAGTCTGTCCATCACGCAAATTAACCGTT
C33	CGCGTCTGATAGGAACGCCATCAACTTTTACA
C34	AGGAAGATGGGGACGACGACAGTAATCATATT
C35	CTCTAGAGCAAGCTTGCATGCCTGGTCAGTTG
C36	CCTTCACCGTGAGACGGGCAACAGCAGTCACA
C37	CGAGAAAGGAAGGGAAGCGTACTATGGTTGCT
C38	GCTCATTTTTTAACCAGCCTTCCTGTAGCCAGGCATCTGC
C39	CAGTTTGACGCACTCCAGCCAGCTAAACGACG
C40	GCCAGTGCGATCCCCGGGTACCGAGTTTTTCT
C41	TTTCACCAGCCTGGCCCTGAGAGAAAGCCGGCGAACGTGG
C42	GTAACCGTCTTTCATCAACATTAAAATTTTTGTTAAATCA
C43	ACGTTGTATTCCGGCACCGCTTCTGGCGCATC
C44	CCAGGGTGGCTCGAATTCGTAATCCAGTCACG
C45	TAGAGCTTGACGGGGAGTTGCAGCAAGCGGTCATTGGGCG
C46	GTTAAAATTCGCATTAATGTGAGCGAGTAACACACGTTGG
C47	TGTAGATGGGTGCCGGAAACCAGGAACGCCAG
C48	GGTTTTCCATGGTCATAGCTGTTTGAGAGGCG
C49	GTTTGCGTCACGCTGGTTTGCCCCAAGGGAGCCCCCGATT
C50	GGATAGGTACCCGTCGGATTCTCCTAAACGTTAATATTTT
C51	AGTTGGGTCAAAGCGCCATTCGCCCCGTAATG
C52	CGCGCGGGCCTGTGTGAAATTGTTGGCGATTA
C53	CTAAATCGGAACCCTAAGCAGGCGAAAATCCTTCGGCCAA
C54	CGGCGGATTGAATTCAGGCTGCGCAACGGGGGATG
C55	TGCTGCAAATCCGCTCACAATTCCCAGCTGCA
C56	TTAATGAAGTTTGATGGTGGTTCCGAGGTGCCGTAAAGCA
C57	TGGCGAAATGTTGGGAAGGGCGAT
C58	TGTCGTGCACACAACATACGAGCCACGCCAGC
C59	CAAGTTTTTTGGGGTCGAAATCGGCAAAATCCGGGAAACC
C60	TCTTCGCTATTGGAAGCATAAAGTGTATGCCCGCT
C61	TTCCAGTCCTTATAAATCAAAAGAGAACCATCACCCAAAT

C62	GCGCTCACAAGCCTGGGGTGCCTA
C63	CGATGGCCCACTACGTATAGCCCGAGATAGGGATTGCGTT
C64	AACTCACATTATTGAGTGTTGTTCCAGAAACCGTCTATCAGGG
C65	ACGTGGACTCCAACGTCAAAGGGCGAATTTGGAACAAGAGTCC
Link-A1C	TTAATTAATTTTTTACCATATCAAA
Link-A2C	TTAATTTCATCTTAGACTTTACAA
Link-A3C	CTGTCCAGACGTATACCGAACGA
Link-A4C	TCAAGATTAGTGTAGCAATACT
Link-B1A	TGTAGCATTCCTTTTATAAACAGTT
Link-B2A	TTTAATTGTATTTCCACCAGAGCC
Link-B3A	ACTACGAAGGCTTAGCACCATTA
Link-B4A	ATAAGGCTTGCAACAAAGTTAC
Link-C1B	GTGGGAACAAATTTCTATTTTTGAG
Link-C2B	CGGTGCGGGCCTTCCAAAAACATT
Link-C3B	ATGAGTGAGCTTTTAAATATGCA
Link-C4B	ACTATTAAAGAGGATAGCGTCC
Loop	GCGCTTAATGCGCCGCTACAGGGC

Table S7. Immunofluorescence antibodies

Primary Antibody	Source/Isotype	Supplier	Catalogue Number	Dilution
10D5	Mouse	Made in house		1:10
Anti-EGFR antibody [EP38Y]	Rabbit	Abcam	ab52894	1:250
ErbB2 (HER-2)	Rabbit	Thermofisher	MA5-15050	1:400
Met (D1C2) XP®Rabbit mAb	Rabbit	CST	8198	1:1500
Phospho-EGF Receptor (Tyr1045) Antibody	Rabbit	CST	2237	1:200
Phospho-HER2/ErbB2 (Tyr1221/1222) (6B12) Rabbit mAb	Rabbit	CST	2243	1:200
Phospho-Met (Tyr1234/1235) (D26) XP® Rabbit mAb	Rabbit	CST	3077	1:800
Phospho-FAK (Tyr397) Antibody	Rabbit	CST	3283	1:100
Phospho-Akt (Ser473)	Rabbit	CST	9271	1:200
Phospho-p44/42 MAPK (Erk1/2) (Thr202/Tyr204) Antibody	Rabbit	CST	9101	1:200

Table S8. Immunoblotting antibodies

Primary Antibody	Source	Supplier	Catalogue Number	Dilution	kDa
Integrin beta6 (0.2 mg/mL)	Goat	Bio-techne	AF2389	1:1000	100
Anti-EGFR antibody [EP38Y]	Rabbit	Abcam	ab52894	1:1000	100
ErbB2 (HER-2)	Rabbit	Thermofisher	MA5-15050	1:1000	185
Met (D1C2) XP® Rabbit mAb	Rabbit	CST	8198	1:1000	140,170
β-Actin	Mouse	Abcam	ab8226	1:1000	42

References

(1) Saha, A.; Ellison, D.; Thomas, G. J.; Vallath, S.; Mather, S. J.; Hart, I. R.; Marshall, J. F. High-Resolution in Vivo Imaging of Breast Cancer by Targeting the pro-Invasive Integrin Avβ6. *The Journal of Pathology* **2010**, *222* (1), 52–63.

(2) Wang, D.-L.; Song, Y.-L.; Zhu, Z.; Li, X.-L.; Zou, Y.; Yang, H.-T.; Wang, J.-J.; Yao, P.-S.; Pan, R.-J.; Yang, C. J.; Kang, D.-Z. Selection of DNA Aptamers against Epidermal Growth Factor Receptor with High Affinity and Specificity. *Biochemical and Biophysical Research Communications* **2014**, *453* (4), 681–685.

(3) Mahlknecht, G.; Maron, R.; Mancini, M.; Schechter, B.; Sela, M.; Yarden, Y. Aptamer to ErbB-2/HER2 Enhances Degradation of the Target and Inhibits Tumorigenic Growth. *Proceedings of the National Academy of Sciences* **2013**, *110* (20), 8170–8175.

(4) Ueki, R.; Sando, S. A DNA Aptamer to C-Met Inhibits Cancer Cell Migration. *Chemical Communications* **2014**, *50* (86), 13131–13134.

(5) Rothemund, P. W. K. Folding DNA to Create Nanoscale Shapes and Patterns. *Nature* **2006**, *440* (7082), 297–302.
